# Validation of a model of rheumatoid arthritis using mice reconstituted with patient peripheral blood mononuclear cells

**DOI:** 10.1242/dmm.052294

**Published:** 2025-12-29

**Authors:** Paula Schuster-Winkelmann, Veronika Weß, Marietta Schindler, Morten Ø. Jensen, David E. Shaw, Paolo Alberton, Hendrik Schulze-Koops, Silvia Schoenthaler, Andreas Weinhaeusel, Matthias Siebeck, Roswitha Gropp, Attila Aszodi

**Affiliations:** ^1^ Department of General, Visceral and Transplantation Surgery, Hospital of the LMU, LMU Munich, 80336 Munich, Germany; ^2^ D. E. Shaw Research, New York, NY 10036, USA; ^3^ Department of Biochemistry and Molecular Biophysics, Columbia University, New York, NY 10032, USA; ^4^ Department for Orthopaedics and Trauma Surgery Musculoskeletal University Center Munich (MUM), LMU University Hospital, LMU Munich, 81377 Munich, Germany; ^5^ Division of Hand, Plastic and Aesthetic Surgery, LMU University Hospital, LMU Munich, 81377 Munich, Germany; ^6^ Department of Med. IV, Hospital of the LMU, LMU Munich, 80336 Munich, Germany; ^7^ Center for Health and Bioresources, Molecular Diagnostics, Austrian Institute of Technology GmbH (AIT), 1210 Wien, Austria

**Keywords:** Rheumatoid arthritis, Humanized mouse model, Autoimmune disease, NOD-*scid* IL2Rγ^null^ mice, Therapeutic evaluation, Inflammatory markers

## Abstract

Rheumatoid arthritis (RA) is a chronic autoimmune disease characterized by inflammation and joint destruction. Replicating human manifestations of RA in animal models remains challenging, however, owing to heterogeneity of the disease. In this study, a humanized mouse model for RA was developed and validated using NOD-*scid* IL2Rγ^null^ (NSG) mice engrafted with peripheral blood mononuclear cells (PBMCs) from patients with RA (NSG-RA). RA symptoms were induced using lipopolysaccharide and a cocktail of antibodies against type II collagen. Pathological manifestations were assessed through clinical scoring of hind paw swelling, histological analysis, and evaluation of RA-specific markers in plasma and joints using Luminex, RT-PCR and RNA sequencing. NSG-RA mice exhibited increased levels of RA-specific markers, an influx of inflammatory cells into the synovium, bone erosion and elevated levels of human autoantibodies. Enriched RNA-sequencing pathway analysis revealed activation of the RA disease pathway, along with the TNF and IL-17 signalling pathways. Treatment with prednisolone or infliximab ameliorated disease symptoms and decreased levels of inflammatory markers. These findings indicate that the NSG-RA model offers a translational tool for studying RA pathogenesis and testing novel therapeutic approaches.

## Teaser

**Summary:** A humanized NOD-*scid* IL2Rγ^null^ mouse model engrafted with peripheral blood mononuclear cells from a patient with rheumatoid arthritis mirrors rheumatoid arthritis pathology and treatment response, enabling translational studies of disease mechanisms and novel therapies.

## INTRODUCTION

Rheumatoid arthritis (RA) is a chronic inflammatory disorder characterized by synovitis primarily affecting the small diarthrodial joints of the hands and feet (Asquith et al., 2009). It is defined by the infiltration of inflammatory cells into the synovium, which leads to proliferation of synovial fibroblasts (SFs) and pannus formation, a process that occurs at the synovial tissue–cartilage junction of the joint and contributes to cartilage and bone destruction (Karouzakis et al., 2006; Furuzawa-Carballeda et al., 2008). These pathological manifestations cause severe pain, stiffness and joint deformation, and can result in disability and reduced life expectancy (Turesson et al., 2002). The most significant genetic risk factors for RA include variations in human leukocyte antigen (HLA) genes, *STAT4*, *TRAF1-C5* and *PTPN22*, all of which are involved in activation of B- and T-cells (Lee et al., 2009). A combination of genetic and environmental factors leads to an inflammatory response, which ultimately leads to chronic inflammation of the joints. Although the initial symptoms emerge locally, RA eventually develops into a systemic disease (Turesson et al., 2002; Asquith et al., 2009).

Standard pharmacological care for RA includes treatment with methotrexate, TNFα (also known as TNF) inhibitors (e.g. adalimumab, infliximab, etanercept), glucocorticoids (e.g. prednisolone), B- and T-cell inhibitors (e.g. abatacept, rituximab), and interleukin (IL)-1, -6 and -17 blockers (such as anakinra, tocilizumab, secukinumab), all of which cause severe side effects due to their immunosuppressive mode of action (Rein and Mueller, 2017). As a consequence, opportunistic infections often require interruption of the therapy. In addition, the treatment regimen often relies on a trial-and-error dose-escalating strategy owing to the lack of diagnostic markers that predict response to therapy. In order to validate potential therapeutic molecules *in vivo*, animal models that recapitulate the clinical and major pathological phenomena of RA are needed.

For decades, numerous rodent models have played a crucial role in investigating the immunopathogenesis of RA and testing potential therapies. These models induce RA symptoms through adjuvant, collagen or proteoglycan application, or genetic modification (Hegen et al., 2008; Bevaart et al., 2010; Vincent et al., 2012; Schinnerling et al., 2019). They have led to important insights, highlighting the role of CD4^+^ T-cells and B-cells, identifying TNFα as a key effector inflammatory cytokine (Hegen et al., 2008; Bevaart et al., 2010; Vincent et al., 2012) and enabling the discovery of various autoantigens (Schinnerling et al., 2019). However, they are often unable to recapitulate human RA etiopathogenesis, which arises from a genetically diverse patient population experiencing long-term disease. Moreover, in these models, the inflammatory responses are mediated exclusively by the murine immune system, making it challenging to translate the findings to human patients (Mestas and Hughes, 2004; Schinnerling et al., 2019). The increasing demand for humanized models has led to various strategies to create mouse models that accurately mimic human disease. One approach involves the transgenic expression of human molecules, such as HLA class II molecules or RA-associated autoantigens, in immunocompetent mice, while other strategies, including the generation of mouse/human chimeras, aim to mimic aspects of the human immune response, such as local inflammation and fibroblast activation (Schinnerling et al., 2019).

For example, NSG mice engrafted with human CD34^+^ umbilical cord blood stem cells and injected with complete Freund's adjuvant developed arthritis, with human immune cells migrating to the injection sites (Misharin et al., 2012). Similarly, NOG mice reconstituted with human CD34^+^ stem cells and infected with Epstein-Barr virus (EBV) developed erosive arthritis, synovial proliferation and pannus formation, suggesting a potential role for EBV in RA (Kuwana et al., 2011). Additionally, NOD/LtSzscid/IL2Rγ^−/−^ mice engrafted with patient-derived B-cells and memory CD4^+^ T-cells produced IgM-rheumatoid factor and anti-aminoacyl transfer RNA synthetase antibodies, making them suitable for autoimmune studies (Ishikawa et al., 2014). Other models, such as the transfer of stimulated synovial fluid T-cells into severe combined immunodeficiency (SCID) mice, induced severe arthritis and are useful for studying RA pathogenesis (Sakata et al., 1996).

In recent years, we have developed humanized mouse models for ulcerative colitis (UC) and Crohn's disease (CD) using NOD-*scid* IL2Rγ^null^ (NSG) mice, the compromised immune systems of which have been reconstituted with peripheral blood mononuclear cells (PBMCs) of patients with the respective diseases (Jodeleit et al., 2017, 2020; Unterweger et al., 2021b). These models recapitulate human disease symptoms and retain aspects of the donor's immunological profile. In this study, we applied the same approach to develop a model for RA. The NSG-RA mouse model is based on the engraftment of unfractionated RA PBMCs, which can be easily obtained via blood draw and injected directly into recipient mice. This confers to the mice a diversity of patient-derived immune cells, including autoreactive T- and B-cells, thus in part mimicking the human immune response, including potential donor variation. NSG-RA mice reflected disease-specific manifestations, such as pathological changes of the joints and altered expression of inflammatory markers known to be correlated with clinical symptoms of RA. Therefore, we believe that this model enables a direct link between donor immune profiles and the disease phenotype, offering a practical, scalable and translationally relevant tool for studying RA pathogenesis and evaluating human-targeted therapies.

## RESULTS

### Inflammatory response in NSG-RA mice is dependent on challenge and immunological profile

In order to elucidate whether an NSG-RA model would be reflective of the human disease, NSG mice reconstituted with PBMCs from five patients with RA and one unaffected individual were compared. All donors had a clinical activity score between 6 and 10 according to American College of Rheumatology (ACR)/European Alliance of Associations for Rheumatology (EULAR) classification (see Table S1 for the complete dataset). Ten days post reconstitution, the mice were divided into two groups: one group was challenged with mouse anti-type II collagen 5-clone monoclonal antibody cocktail (anti-col II) and the other was left unchallenged. Three days post anti-col II injection, lipopolysaccharide (LPS) was applied to the challenged group to trigger rapid onset of inflammation and increase the severity of arthritis. To further exacerbate the arthritis symptoms, and thereby help to ensure that disease development could be clearly observed, the application scheme was repeated after 7 days. Mice reconstituted with PBMCs from patients with RA are referred to as the NSG-RA unchallenged and NSG-RA challenged groups, whereas mice reconstituted with PBMCs from an unaffected individual are referred to as the NSG-nonRA unchallenged and NSG-nonRA challenged groups. The onset of disease was assessed by monitoring murine hind paw swelling and weight loss (see scoring system in Table S2), followed by histological examination of the interphalangeal and metatarsophalangeal joints, analysis of human leucocyte sub-populations isolated from the spleen, measurement of inflammatory markers in the plasma and joint tissue, and detection of autoantibodies.

**Fig. 1.**
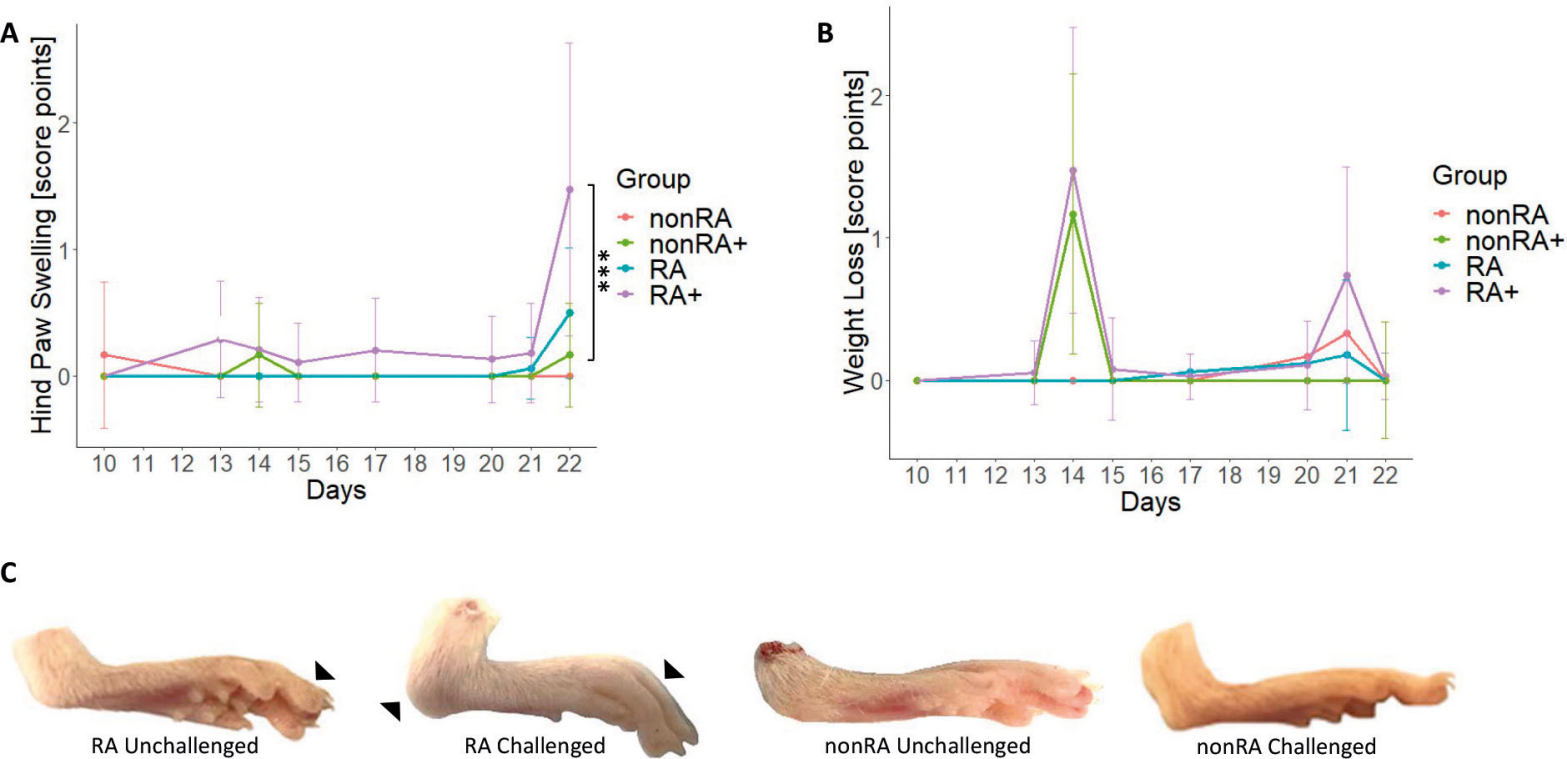
**Immunological profile had an impact on frequencies of hind paw swellings *in vivo*.** (A,B) Hind paw swelling (A) and body weight loss (B) of the mice were monitored over time and shown as score point mean±s.d. in a scatter plot. Mice were reconstituted with peripheral blood mononuclear cells (PBMCs) from patients with rheumatoid arthritis (RA) (RA3, *n*=11; RA4, *n*=6; RA5, *n*=11; RA7, *n*=6; RA8, *n*=15) and an unaffected individual (RA6, *n*=12) and were left unchallenged (indicated as ‘RA’ and ‘nonRA’) or challenged with mouse anti-type II collagen antibody on days 10 and 17 and with lipopolysaccharide (LPS) on days 13 and 20 (indicated as ‘RA+’ or ‘nonRA+’). *N*=number of donors, *n*=total number of mice. RA, *N*=3, *n*=17; RA+, *N*=5, *n*=32; nonRA, *N*=1, *n*=6; nonRA+, *N*=1, *n*=6. Asterisks indicate statistically significant differences between groups on day 22 (***P≤0.001). Statistical analysis was performed with R (two-way ANOVA). (C) Representative macrophotographs of mouse hind paws. Arrowheads indicate swelling in interphalangeal joints of the toes and the ankle joint.

Following challenge on days 10+13 and 17+20, NSG-RA mice exhibited swelling of the hind paws and decreased body weight, whereas unchallenged NSG-RA mice rarely developed these symptoms, leading to significant differences in hind paw swelling between the two groups (Fig. 1A, *P*=0,003; for complete dataset, see Table S7). Animals reconstituted with PBMCs from a healthy (nonRA) donor displayed no significant increase in hind paw swelling, regardless of challenge, with the exception of weight loss observed following LPS administration. In contrast, on day 22, the incidence of hind paw swelling was significantly higher in challenged NSG-RA mice compared to that in challenged NSG-nonRA mice (Fig. 1A, *P*=0,01). Representative macrophotographs clearly identify swelling of the ankle joint and interphalangeal joints in NSG-RA mice, although not in the NSG-nonRA mice, indicating that this model is strongly dependent on the immunological profile (Fig. 1C).

**Fig. 2.**
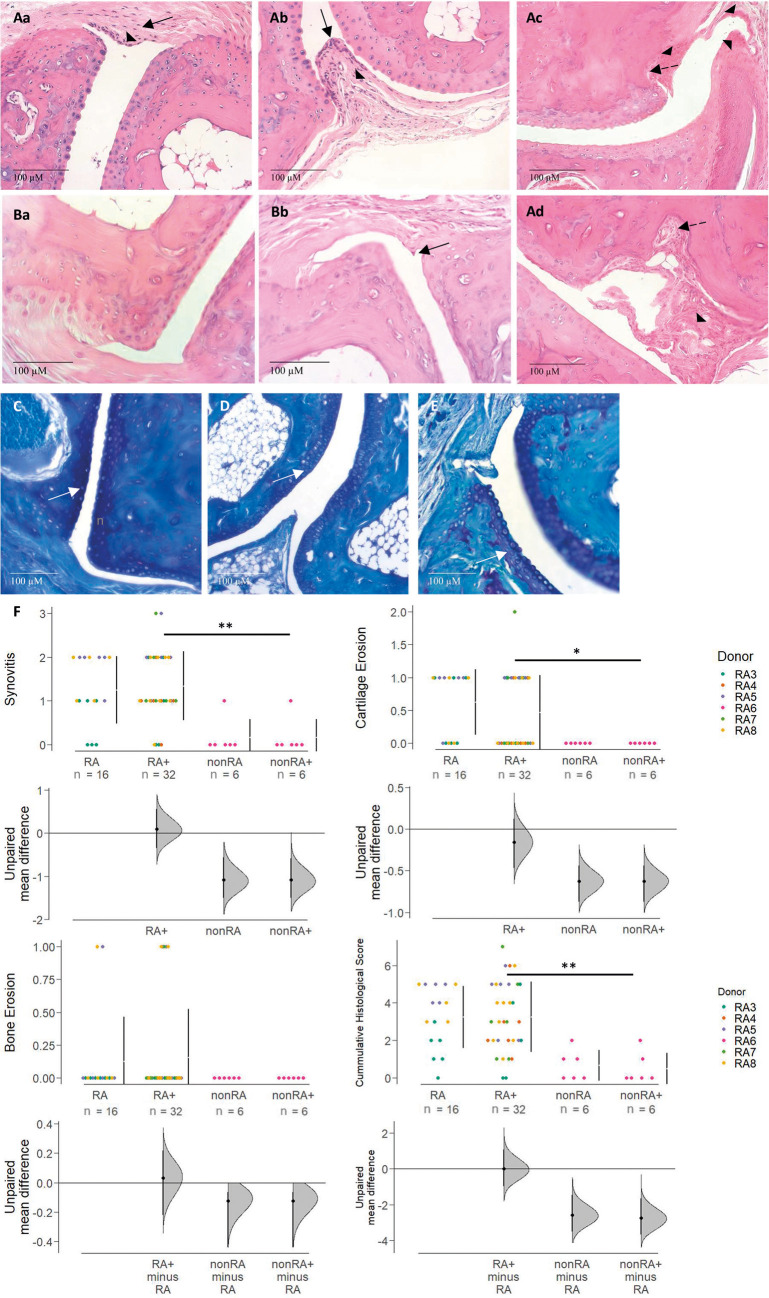
**Histopathological changes in response to anti-type II collagen antibody and LPS challenge were dependent on the immunological background of the donor.** Mice were treated as described in Fig. 1. (Aa-Bb) Histological sections (20-fold magnification) of murine middle and proximal interphalangeal joints stained with Haematoxylin and Eosin (H&E) and Toluidine Blue (TB). Aa, Ab, Ac and Ad present joints of mice reconstituted with PBMCs of patients with RA. Aa shows a section from an unchallenged NSG-RA mouse [NOD-*scid* IL2Rγ^null^ (NSG) mouse engrafted with PBMCs from a patient with RA], whereas Ab and Ac show sections from a challenged NSG-RA mouse. Thickening of the intimal layer and pannus formation are marked by arrowheads. Influx of inflammatory cells into the synovium is indicated by solid line arrows, and signs of bone erosion are indicated by dashed line arrows. Ba and Bb present microphotographs of H&E-stained sections from mice reconstituted with PBMCs from an unaffected individual (Ba, unchallenged; Bb, challenged; arrow in Bb is representative of inflammatory cell influx in both NSG-nonRA groups). (C-E) TB staining of a challenged mouse reconstituted with PBMCs from an unaffected individual (C) and challenged mice reconstituted with PBMCs of patients with RA (D,E). White arrows indicate proteoglycan degradation. (F) Histological scores for synovitis, cartilage erosion, bone erosion and the cumulative histological score, depicted as Cumming plots (RA, *N*=3, *n*=16; RA+, *N*=5, *n*=32; nonRA and nonRA+, *N*=1, *n*=6). *N*=number of donors, *n*=total number of mice. Mice were reconstituted with PBMCs from patients with RA (RA3, *n*=11; RA4, *n*=6; RA5, *n*=11; RA7, *n*=6; RA8, *n*=15) and a healthy individual (RA6, *n*=12). **P≤0.01, *P≤0.05. Statistical analysis was performed with R (two-way ANOVA).

To further corroborate these observations, histological examination of murine hind paws was performed. Haematoxylin and Eosin (H&E)- and Toluidine Blue (TB)-stained sections of medial and proximal interphalangeal and metatarsophalangeal joints from challenged NSG-RA mice revealed that anti-col II and LPS evoked morphological changes in the joints. These included synovia characterized by hyperplasia of the intima synovialis and influx of inflammatory cells into the intima and subintima (Fig. 2Ab). Additionally, the formation of invasive panni and bone erosion at the bare areas was observed (Fig. 2Ac,Ad). Roughening of the cartilage surface indicated cartilage erosion. Of note, reconstitution with PBMCs from patients with RA was sufficient (i.e. without challenge) to induce histomorphological changes in the joints, as presented by an influx of inflammatory cells into the synovium and moderate thickening of the intima synovialis (Fig. 2Aa). TB staining revealed that mice reconstituted with healthy donor PBMCs (Fig. 2C) retained intense metachromatic staining of articular cartilage, indicating preserved proteoglycan content and intact cartilage structure. In contrast, mice receiving PBMCs from patients with RA (Fig. 2D,E) showed markedly reduced TB staining, indicative of proteoglycan loss and cartilage degradation. Histological examination of NSG-nonRA mice did not show any signs of arthritis, regardless of challenge, and the histological scores also trended lower than those of the unchallenged NSG-RA control group (Fig. 2F). However, occasional infiltration of inflammatory cells was identifiable in both NSG-nonRA groups (representatively exemplified in Fig. 2Bb). As depicted in Fig. 2F, the histological scores, with the exception of bone erosion, were significantly higher in both NSG-RA groups compared to those in the NSG-nonRA groups, highlighting the preservation of the immunological profile in this model. NSG-RA mice displayed histopathological signs of RA, and subsequent antibody challenge did not further enhance these signs in the joints of these mice.

**Fig. 3.**
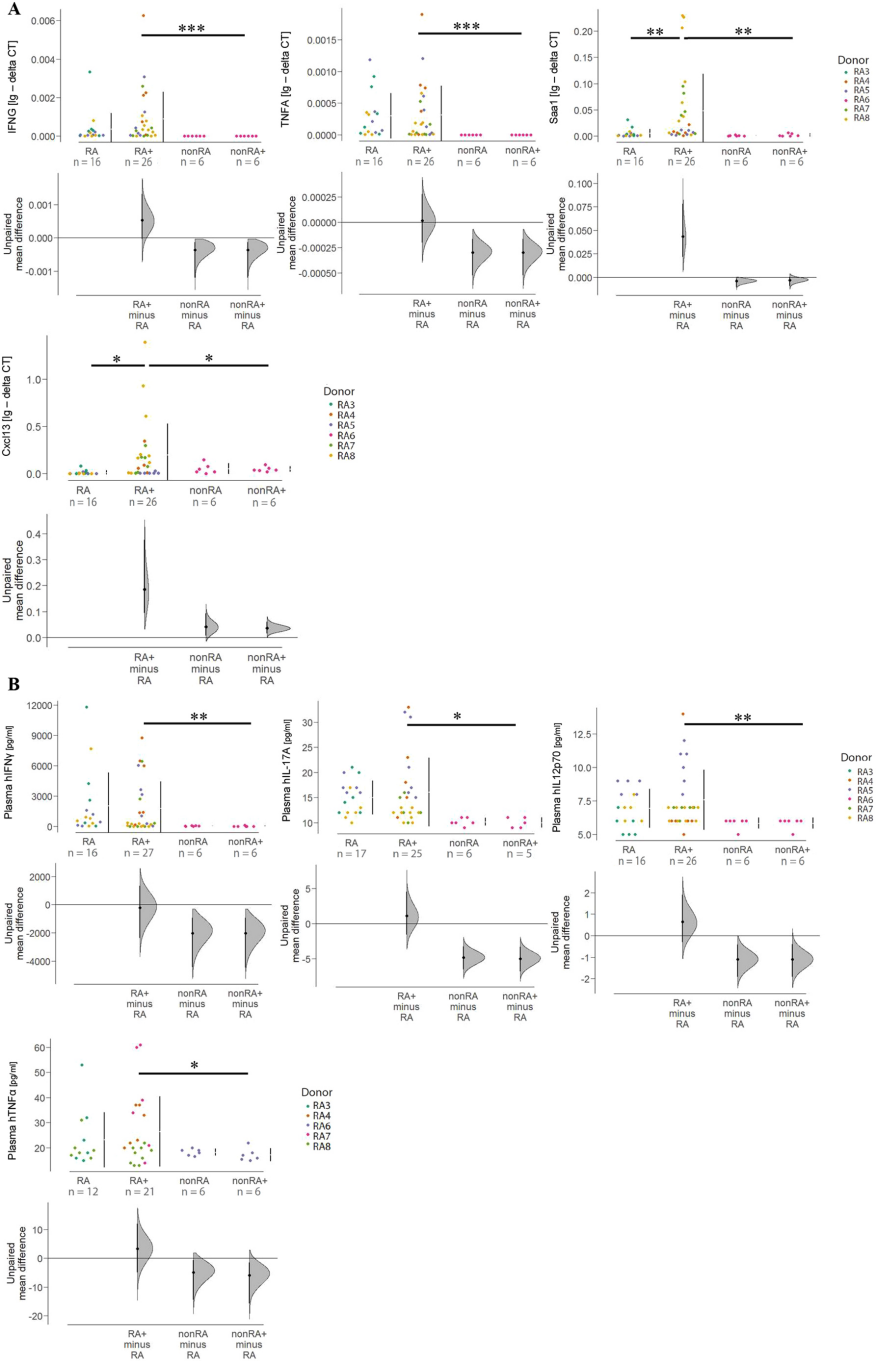
**Human and mouse inflammatory markers in arthritic joints and plasma are increased in NSG-RA mice.** Mice were treated as described in Fig. 1. (A) RT-PCR expression analysis of *IFNG*, *TNFA*, *Saa1* and *Cxcl13* depicted as Cumming plots. CT values over 40 cycles and undetermined values were considered as not expressed and set to zero. (B) Plasma expression levels of human IFNγ, IL-17A, IL12p70 and TNFα detected by Luminex assay and displayed as Cumming plots. The upper part of the plot presents each data point in a swarmplot. The mean and s.d. of each group is plotted as a gapped line, where the vertical lines correspond to the mean±s.d. and the mean itself is depicted as a gap in the line (***P≤0.001, **P≤0.01, *P≤0.05). Statistical analysis was performed with R (Kruskal–Wallis test). mRNA levels are depicted as lg(−deltaCT). RA, *N*=3, *n*=17; RA+, *N*=4, *n*=27; nonRA, *N*=1, *n*=6; nonRA+, *N*=1, *n*=6. Variations in sample sizes can be attributed to challenges encountered during RNA isolation and the exclusion of outliers during data processing. Values deviating more than sixfold from the mean were considered outliers. As indicated in the key to the right of the lower plot in B, plasma from the RA5 samples was not included in the human TNFα analysis, resulting in a decreased sample size for that plot. *N*=number of donors, *n*=total number of mice. Mice were reconstituted with PBMCs from patients with RA (RA3, *n*=6; RA4, *n*=6; RA5, *n*=11; RA7, *n*=6; RA8, *n*=15) and an unaffected individual (RA6, *n*=12).

To gain a more detailed understanding of the inflammatory responses, we conducted RNA-sequencing (RNA-seq) analysis of mRNA isolated from murine hind paws of unchallenged (*N*=2, *n*=6 in total) and challenged (*N*=3, *n*=6 in total) NSG-RA mice. Samples were analysed for differential expression of mouse and human genes (see Tables S8 and S9 for complete dataset). The analysis of human genes revealed that 366 genes were upregulated with |log2 (FoldChange)|≥1 and *P*<0.05, including *IFNG* (encoding interferon gamma) and *CXCL13* (encoding C-X-C motif chemokine 13), both of which are associated with RA (Bechman et al., 2020; Kato, 2020) (Fig. S2A and Table S8). Disease ontology analysis revealed that arthritis and RA are significantly associated with the differentially expressed genes (Fig. S2B). The analysis of murine genes revealed 489 upregulated genes with |log2 (FoldChange)|≥1 and *P*<0.05, including *Cxcl13*, matrix metallopeptidase 3 (*Mmp3*) and serum amyloid A1 (*Saa1*), all of which are associated with RA (Vallon et al., 2001; Bechman et al., 2020) (Fig. S3A and Table S9). Based on the pattern of upregulated genes, an enriched Kyoto Encyclopedia of Genes and Genomes (KEGG) analysis identified the RA disease pathway, along with the IL-17 and TNF signalling pathways, as the most significantly activated (Fig. S3B). Pathview analysis identified upregulated genes in the RA disease pathway (Fig. S3C). As the analysis of differential gene expression revealed significant differences solely based on the unadjusted *P*-values, the expression levels of *IFNG*, *TNFA*, *Cxcl13* and *Saa1* within the joints were validated through RT-PCR. Upon challenge, the expression levels of all four markers were increased in NSG-RA mice; however, this increase was significant only for *Cxcl13* and *Saa1*. By way of contrast, these markers were hardly detectable in NSG-nonRA mice, regardless of the challenge. The differences in expression levels were significant for all four markers, when NSG-nonRA challenged mice were compared to NSG-RA challenged mice (Fig. 3A). For the analysis of cytokine levels in murine plasma, Luminex assays were performed. The concentration of the human cytokines IFNγ, IL-17A, IL12p70 and TNFα were measured and compared (Fig. 3B). The pattern of this analysis was similar to the RT-PCR analysis. Challenge of the NSG-RA mice did not affect the levels of these cytokines; levels of these four cytokines were all significantly different when comparing the two challenged groups (NSG-RA and NSG-nonRA; Fig. 3B).

**Fig. 4.**
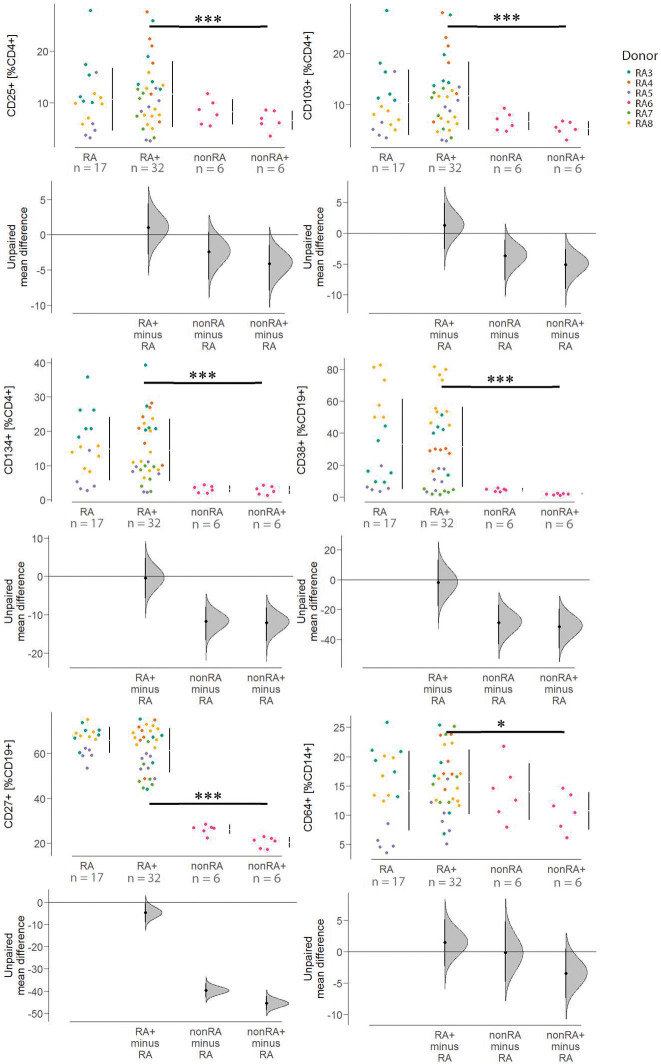
**Reconstitution with PBMCs from unaffected or RA-diagnosed individuals had differing impacts on the expression of human inflammatory leucocyte frequencies *in vivo*.** Mice were treated as described in Fig. 1. Leucocytes were isolated from murine spleen, and cell type frequencies were analysed by flow cytometry and depicted as Cumming plots. The upper part of the plot presents each data point in a swarmplot. The mean and s.d. of each group is plotted as a gapped line, where the vertical lines correspond to the mean±s.d., and the mean itself is depicted as a gap in the line (***P≤0.001, *P≤0.05). Statistical analysis was performed with R (Kruskal–Wallis test). RA, *N*=3, *n*=17; RA+, *N*=5, *n*=32; nonRA, *N*=1, *n*=6; nonRA+, *N*=1, *n*=6. *N*=number of donors, *n*=total number of mice. For values, see Table S7. Mice were reconstituted with PBMCs from patients with RA (RA3, *n*=11; RA4, *n*=6; RA5, *n*=17; RA7, *n*=6; RA8, *n*=15) and an unaffected individual (RA6, *n*=12).

To examine the effect of the immunological profile and challenge on the frequencies of different inflammatory cell types, flow cytometric analysis of human splenic leucocytes was performed (for definition of cell types, see Table S5). The flow cytometry revealed no significant differences between the NSG-RA unchallenged and NSG-RA challenged groups, but we observed low frequencies of all analysed cell types in the NSG-nonRA mice regardless of the challenge (Fig. 4).

The differences between the NSG-nonRA challenged and the NSG-RA challenged mice were significant. This observation applied to the frequencies of activated T-cells (CD4^+^CD25^+^, CD4^+^CD103^+^ and CD4^+^CD134^+^), experienced B-cells (CD19^+^CD27^+^) and plasma B-cells (CD19^+^CD38^+^). One possible explanation for the requirement of an RA inflammatory background and the spontaneous development of RA symptoms in the NSG-RA model could be the impact of autoreactive T- and B-cells from the patients with RA. To examine the autoimmune background in the NSG-RA model, we used protein microarrays to analyse the levels of human autoantibodies. Human autoantibodies from the following groups were compared: NSG-nonRA unchallenged (*N*=1, *n*=6 in total), NSG-nonRA challenged (*N*=1, *n*=6 in total), NSG-RA unchallenged (*N*=2, *n*=11 in total) and NSG-RA challenged (*N*=2, *n*=12 in total). Plasma was collected on the last day of the study. We identified 194 autoantibodies with significant differences between the NSG-nonRA unchallenged and challenged groups (significance for the univariate test was set at *P*<0.05). The number of differentially expressed autoantibodies increased to 799 when the NSG-RA unchallenged and challenged groups were compared and, as anticipated, was even higher when comparing the NSG-nonRA and NSG-RA challenged groups (1274 autoantibodies, see Table S10). The presence of human autoantibodies in mouse plasma suggested that the expression of these antibodies resulted from the PBMC engraftment transferring human B- and T-cells onto these mice. Autoantibodies recognizing various types of collagen were among those that were differentially regulated. KEGG pathway analysis identified significant enrichment of autoantibodies targeting autoantigens associated with focal adhesion (see Fig. S4).

**Fig. 5.**
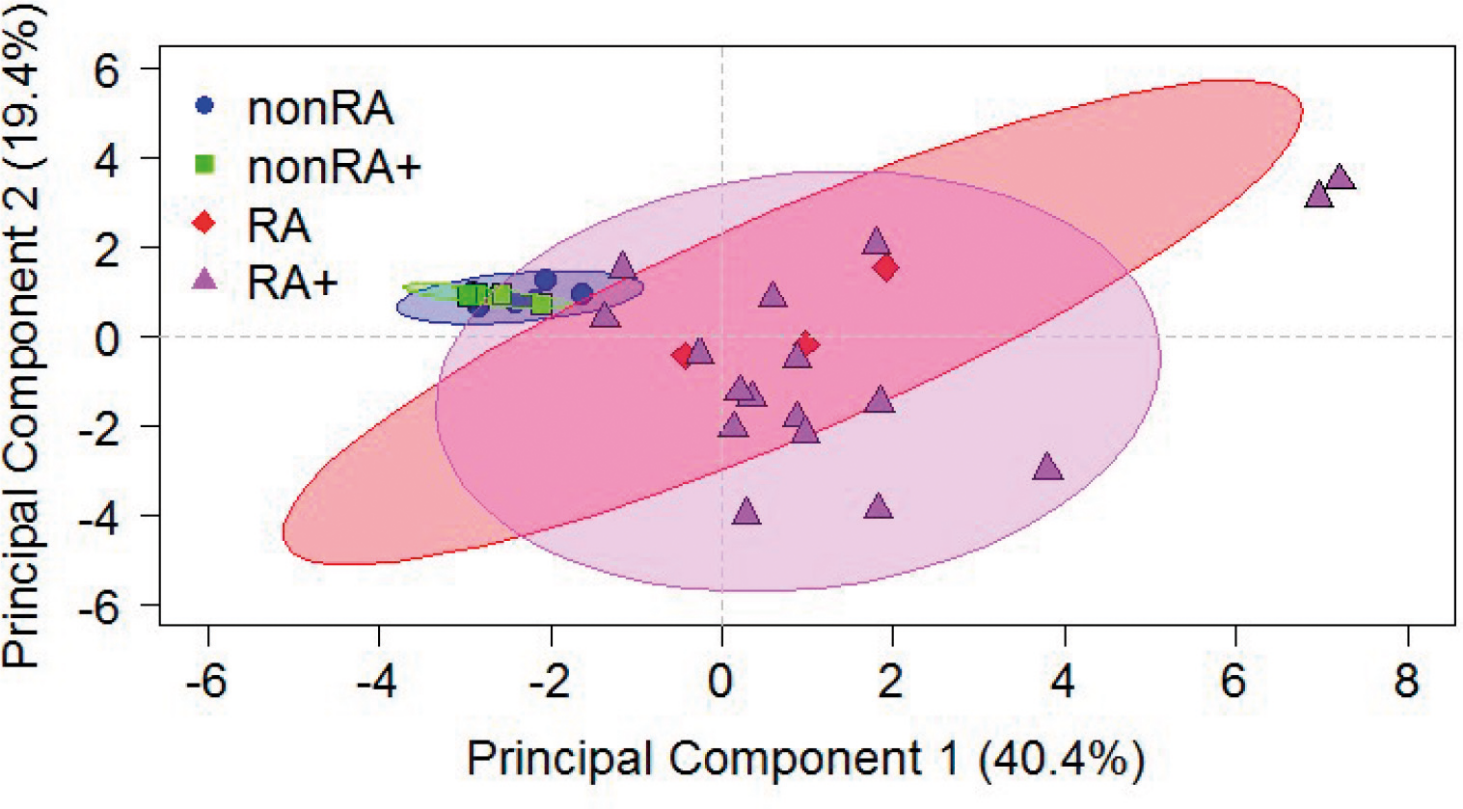
**Principal component analysis corroborates the impact of the immunological profile.** Mice were treated as described in Fig. 1. All *in vivo* scores and fluorescence-activated cell sorting, Luminex and RT-PCR data were used as variables. Ellipses display confidence intervals of 0.95.

To assess the impact of the immunological profile on the NSG-RA phenotype, a principal component analysis (PCA) was performed including all of the scores and markers previously presented. The PCA revealed that NSG-nonRA mice clustered closely together, whereas NSG-RA mice were scattered, independently of challenge, reflecting the heterogeneity and variability observed in this model. This indicated that the development of RA in this model is strongly dependent on the immunological profile of the RA patient, and that the challenge could be used to further exacerbate certain symptoms of arthritis in NSG-RA mice (Fig. 5).

### Characterization of SFs in the NSG-RA mouse model

**Fig. 6.**
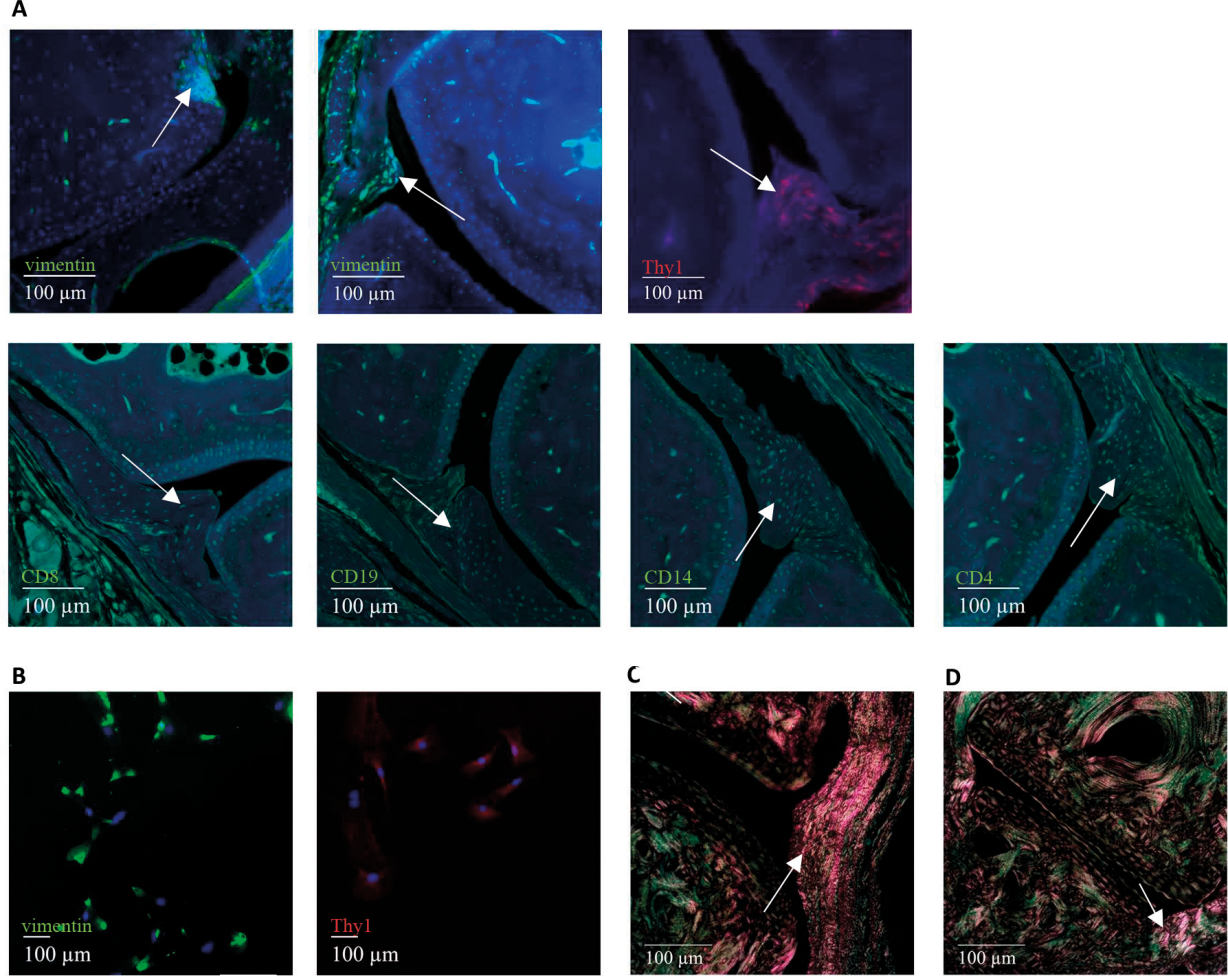
**Characterization of synovial fibroblasts (SFs) in the NSG-RA model.** (A) Mouse vimentin^+^ SFs in the hyperplastic intimal layer of the synovium (upper middle, arrow) and pannus areas (upper left, arrow) of a challenged NSG-RA mouse. Murine Thy1^+^ SFs in the sublining layer of the inflamed synovium (upper right, arrow). Human CD4^+^, CD8^+^, CD14^+^ and CD19^+^ leucocytes infiltrating the hyperplastic synovium (lower row, arrows, 20× magnification). (B) Immunocytochemistry of SFs isolated from challenged NSG-RA mice (10× magnification). Isolated SFs expressed the markers vimentin and Thy1. (C,D) Sirius Red staining under polarized light (20× magnification). Proliferation of synovial sublining and lining fibroblasts increased collagen I and III deposition in inflamed synovium of challenged NSG-RA mice (C), compared to that in challenged NSG-nonRA mice (D).

Elements of fibrotic disease are known to occur with progression of RA from mild to severe disease (Steenvoorden et al., 2006; Wu et al., 2021), so a detailed analysis of murine interphalangeal or knee SFs was performed to assess disease progression in our NSG-RA model. Immunofluorescent staining (IFS) and immunocytochemistry (ICC) experiments were performed to analyse the expression of two key fibroblast markers, vimentin and Thy1 (CD90), along with the leucocyte markers (CD4, CD14, CD8 and CD19). IFS revealed a mixed infiltrate of immune cells including CD4^+^ T-cells, cytotoxic CD8^+^ T-cells, CD14^+^ monocytes and CD19^+^ B-cells into the synovium of the challenged NSG-RA mice (Fig. 6A).

Vimentin^+^ and Thy1^+^ SFs were detected at the sites of intimal hyperplasia and pannus formation (Fig. 6A). Thy1 expression was detectable in the synovial lining and sublining. ICC of cultured SFs isolated from NSG-RA mice confirmed the expression of vimentin and Thy1 *ex vivo* (Fig. 6B). The evaluation of Sirius Red-stained inflammatory sections of challenged NSG-RA mice under polarized light revealed an increase in collagen I (orange, thick fibres) and collagen III (green, thin fibres) deposition in the inflamed synovium compared to that in challenged NSG-nonRA mice (Fig. 6C,D). The analyses indicate that, as in the human disease, hyperproliferative SFs play a driving role in this model and that the model can reflect the human disease.

### Treatment with prednisolone and infliximab ameliorates disease progression in the NSG-RA mouse model

**Fig. 7.**
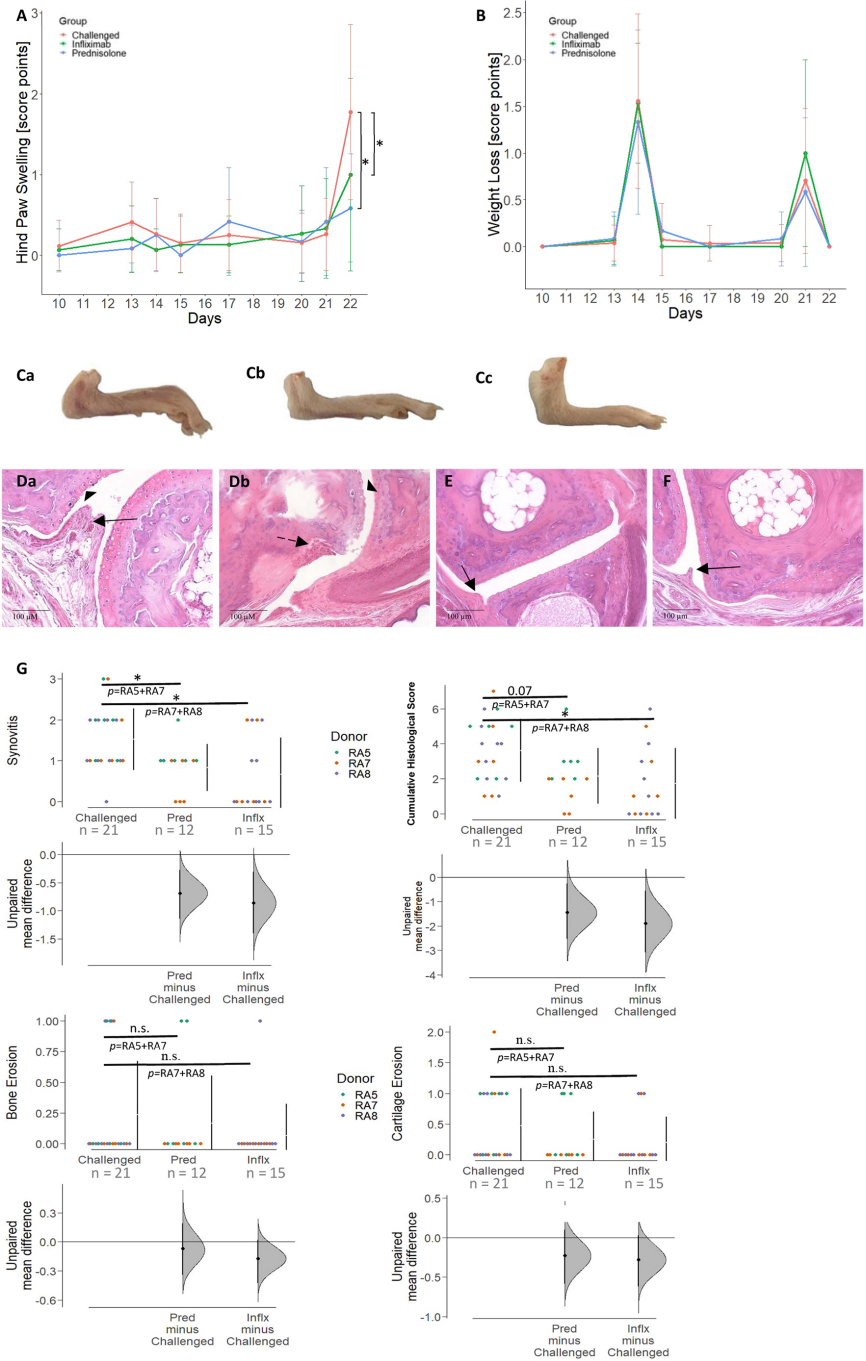
**Prednisolone and infliximab reduced hind paw swelling *in vivo* and histological signs of arthritis.** (A,B) Hind paw swelling (A) and body weight loss (B) were monitored over time and are shown as score point mean±s.d. in scatter plots. NSG mice were reconstituted with PBMCs from patients with RA (*N*=3, *n*=21) and challenged with anti-type II collagen antibody and LPS on days 10/13 and 17/20, respectively. Mice were left untreated or treated with prednisolone (1 mg/kg, *N*=2, *n*=12; administered on days 12, 13, 14, 19, 20 and 21) or infliximab (6 mg/kg, *N*=2, *n*=15; administered on days 12 and 19). Asterisks indicate significant group differences on day 22 [*P≤0.05; R-based analysis (Kruskal–Wallis test)]. (Ca-Cc) Representative images of hind paws from an untreated and challenged NSG-RA mouse (Ca), and challenged NSG-RA mice treated with prednisolone (Cb) or infliximab (Cc). (Da-F) Histological sections of the medial (Da) and proximal (Db) interphalangeal joints from an untreated, challenged NSG-RA mouse, a prednisolone-treated (E) and an infliximab-treated (F) NSG-RA mouse. Solid line arrows indicate inflammatory cell infiltration; dashed line arrows indicate bone erosion; arrowheads indicate cartilage roughening. No hyperplasia, cartilage erosion or bone destruction were seen in treated mice. (G) Cumming plots show histological scores for synovitis, bone erosion, cartilage erosion and the cumulative histological score. Swarmplots depict individual data points; gapped lines represent group mean±s.d. [*P≤0.05; n.s., not significant; R-based analysis (Mann–Whitney *U*-test)]. To account for inter-donor variability, statistical comparisons were made only between treated and challenged mice that received PBMCs from the same RA donors. *N*=number of donors; *n*=total number of mice.

To validate the model, the effects of prednisolone and infliximab treatment on RA disease symptoms were examined. NSG mice were reconstituted with PBMCs from patients with RA and challenged according to the standard protocol described in the Materials and Methods. Infliximab was applied intraperitoneally on days 12 and 19, and prednisolone was applied orally on days 12, 13, 14, 19, 20 and 21. RA symptoms in NSG-RA mice were alleviated by treatment with prednisolone (*P*=0.005) and infliximab (*P*=0.05) treatment, as indicated by reduced hind paw swelling (Fig. 7A). However, administration of LPS led to pronounced weight loss in all groups (Fig. 7B). Representative macrophotographs of murine hind paws treated with prednisolone (Fig. 7Cb) and infliximab (Fig. 7Cc) demonstrate reversion to a healthier appearance of the paws relative to that of untreated mice (Fig. 7Ca). Hind paws were collected post-mortem, processed and stained with H&E for histological analysis. In comparison to the histological analyses of the challenged, untreated NSG-RA mice (Fig. 7Ea,Eb), the histological analyses of prednisolone or infliximab-treated NSG-RA mice revealed significant reduction of synovitis (Fig. 7G), as indicated by a reduced influx of inflammatory cells into the synovium. Scores for bone and cartilage erosion decreased, although not significantly. Histopathological manifestations were classified according to a histological score and confirmed the ameliorating effect of infliximab and prednisolone treatment after challenge (Fig. 7Da-F).

Assessment of synovitis was performed according to a standardized scoring system described in Table S2, evaluating two or more interphalangeal joints per mouse (Hayer et al., 2021). Histological scores for synovitis, cartilage erosion and bone erosion were assessed per mouse, summed accordingly, and presented in separate graphs for each parameter as well as for the cumulative histological score.

**Fig. 8.**
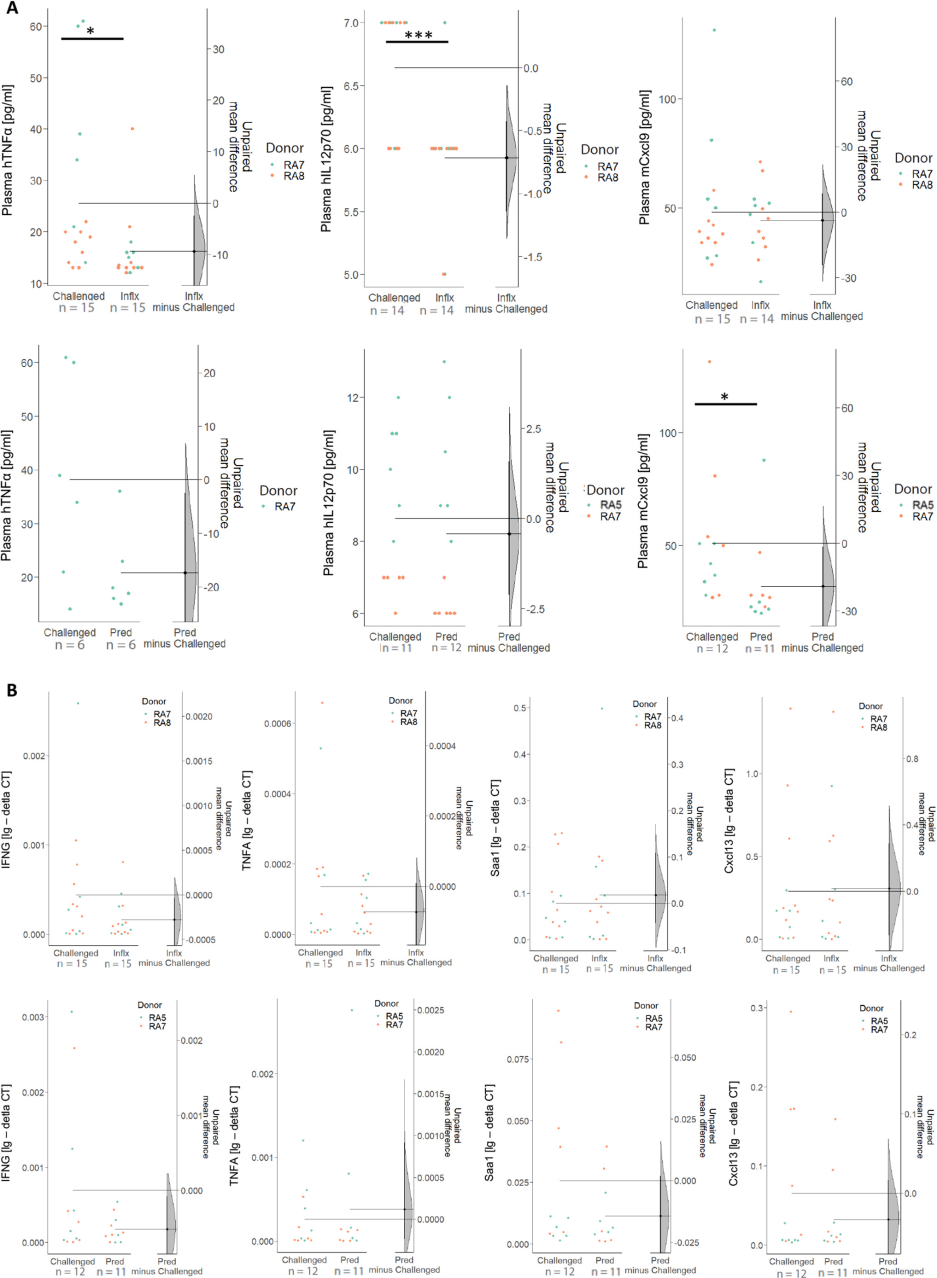
**Cytokine concentration in plasma and expression of human and mouse proinflammatory cytokines in arthritic joints are decreased in response to treatment with prednisolone or infliximab.** Mice were treated as described in Fig. 7. (A) Plasma levels of human TNFα, human IL12p70 and murine Cxcl9 presented as Cumming plots. Challenged, non-treated NSG-RA mice (*N*=2, *n*=15 in total) were compared to challenged, infliximab-treated (*N*=2, *n*=15 in total) or challenged, prednisolone-treated (*N*=2, *n*=12 in total) mice. Outliers (values above 44,000 for Luminex) or undetermined values were removed for data analysis. (B) RT-PCR expression analysis of *TNFA*, *Cxcl13*, *Saa1* and *IFNG* calculated as lg(-deltaCT) and presented as Cumming plots. Challenged, non-treated NSG-RA mice (*N*=3, *n*=21 in total) were compared to challenged, infliximab-treated (*N*=3, *n*=15 in total) or challenged, prednisolone-treated (*N*=2, *n*=11 in total) mice. The upper part of the plot presents each data point in a swarmplot. The mean and s.d. of each group is plotted as a gapped line, where the vertical lines correspond to the mean±s.d., and the mean itself is depicted as a gap in the line (***P≤0.001, *P≤0.05). *N*=number of donors, *n*=total number of mice. Statistical analysis was performed with R (Mann–Whitney *U*-test). For raw data, see Table S7. Mice were reconstituted with PBMCs from patients with RA (RA5, *n*=12; RA7, *n*=18; RA8, *n*=18).

Complementary analyses of plasma cytokine levels by Luminex assays showed that mouse Cxcl9 levels were significantly reduced only by prednisolone treatment (Fig. 8A). In contrast, treatment with infliximab reduced levels of human TNFα in both murine hind paws and plasma, in line with it being an anti-TNFα-based therapy. However, only the reduction in plasma TNFα reached statistical significance. In addition, human IL12p70 levels in mouse plasma were significantly reduced by infliximab treatment (Fig. 8A). mRNA expression analysis was performed on RNA isolated from murine hind paws to further assess treatment effects. RT-PCR quantification of the previously identified markers (*TNFA*, *IFNG*, *Cxcl13* and *Saa1*) revealed that infliximab treatment decreased expression of *TNFA* and *IFNG,* albeit not significantly (Fig. 8B). The expression of mouse *Cxcl13* and *Saa1* was reduced only by prednisolone, which also led to a reduction in *IFNG* levels.

## DISCUSSION

Previous studies have shown that NSG-UC and NSG-CD mice, humanized models for UC and CD, respectively, are highly reflective of their corresponding human diseases (Palamides et al., 2016; Jodeleit et al., 2017, 2020; Unterweger et al., 2021b). Therefore, the first objective of this study was to demonstrate that a similar approach could be used to confer the symptoms and pathophysiological manifestations of RA in an NSG-RA model. For this purpose, PBMCs from donors with RA were used for reconstitution, and the mice were challenged with LPS and a cocktail of antibodies against collagen type II. This procedure has been shown to induce arthritis in BALB/c mice (Nandakumar and Holmdahl, 2007). We also investigated whether an inflammatory stage of RA was required for the induction of symptoms and pathological manifestation, and if the RA background itself was sufficient without additional challenge. The proportion of human CD45^+^ (also known as PTPRC^+^) cells, used as a measure of reconstitution, does not differ between RA and non-RA engrafted mice, highlighting the consistency of the engraftment process across both groups (Table S7). In NSG-RA mice, *in vivo* signs of arthritis as well as the expression of murine Saa1 and Cxcl13 in the joints increased upon challenge, whereas the development of histopathological alterations and the secretion of inflammatory plasma cytokines occurred spontaneously, without challenge. The expression levels of human and murine joint inflammatory markers revealed significant differences between challenged NSG-RA and NSG-nonRA mice, indicating the important role of human leucocytes and challenge in this model; however, only levels of murine Cxcl13 and Saa1 responded to challenge in the NSG-RA mice. Both markers are associated with the initiation of synovial inflammation, either through promoting the development of B-cell follicles and germinal centre reactions (murine Cxcl13) or by activating T-cells (murine Saa1) (Bechman et al., 2020; Leung et al., 2022). Thus, as in the NSG-UC model (Weß et al., 2023), NSG-RA mice might be more sensitive to challenge, but not for all RA disease-relevant inflammatory markers or histological manifestations caused by the inflammation.

Swelling of the hind paw joints seemed to be dependent on both the anti-col II challenge and the LPS challenge, suggesting that this clinical manifestation reflects an acute inflammatory response that may derive from a different mechanism than the synovitis we observed histologically, whereby the presence of RA PBMCs seemed sufficient to induce robust synovial inflammation. The challenge did not amplify the synovitis, but rather appeared to affect other aspects of disease pathology, such as the expression of RA-related genes (*Saa1*, *Cxcl13*). Interestingly, NSG-nonRA mice did not develop arthritis, despite receiving both the anti-col II challenge and the LPS challenge, which are intended to elicit an innate immunity-driven response. One possible explanation for this is that NSG-nonRA mice lack the fully functional and mature, pro-inflammatory or autoreactive immune profile necessary to amplify the inflammatory response. In contrast, NSG-RA mice harbour autoreactive immune cells and human antibodies, likely enhancing susceptibility to the challenge. The PCA analysis emphasizes the importance of the donor background in this model, as the NSG-RA and NSG-nonRA groups formed distinct clusters, and values for the NSG-RA mice increased and dispersed, reflecting the greater immunological heterogeneity observed in NSG-RA mice compared to that in NSG-nonRA mice.

The inflammatory milieu in NSG-RA mice suggests a T-helper (Th)1-dependent inflammation, which corresponds to the classification of RA as a Th1-mediated disease (Bazzazi et al., 2018). Regardless of challenge, NSG-RA mice revealed a pronounced increase of activated T-cells (CD4^+^CD25^+^, CD4^+^CD103^+^ and CD4^+^CD134^+^) in splenic leucocytes. These T-cells are known to be key markers of inflammation in RA, owing to their abundance in the inflamed synovium (Vinay and Kwon, 2012). Furthermore, in mouse joints, the expression of RA-specific inflammatory marker genes encoding human IFNγ and TNFα and murine Cxcl13 and Saa1 was increased considerably in NSG-RA mice compared to that in NSG-nonRA mice, as shown by RT-PCR and RNA-seq analysis (Fig. 3) (Vallon et al., 2001; Vinay and Kwon, 2012; Burska et al., 2014; Lu et al., 2014; Armas-González et al., 2018; Bazzazi et al., 2018; Tardito et al., 2019; Zhang et al., 2019; Bechman et al., 2020; Shapiro, 2021). KEGG enriched pathway analysis of the NSG-RA mouse gene expression identified the TNF signalling pathway, as well as the RA disease pathway, as among the most enriched, indicating RA-specific gene expression. This result was confirmed by disease ontology analysis of the human genome, which also identified RA.

Plasma levels of inflammatory markers known to be clinically relevant for assessing symptoms of RA (Moelants et al., 2013; Burska et al., 2014) (specifically, elevated plasma levels of human IFNγ, TNFα and IL-12) confirmed this pattern and indicate a Th1-mediated inflammatory milieu in NSG-RA mice. Moreover, it has been reported that increased IFNγ production in humans stimulates CXCL9 secretion, and we note that this cytokine was increased in challenged NSG-RA mice (in contrast to NSG-nonRA mice), suggesting the involvement of Th1 lymphocytes (Fig. S5) (Ruschpler et al., 2003). Although Th1 cells have been considered to be the main drivers of inflammation in RA, recent work suggests that Th17 cells also contribute to the severity of RA. Specifically, it has been shown that the overexpression of the IL-17A cytokine induces joint inflammation, bone erosion and loss of cartilage proteoglycan, and directly activates chondrocytes to inhibit and degrade cartilage matrix (Gaffen, 2009; Vinay and Kwon, 2012). These results are consistent with our observation of increased human IL-17A plasma levels in NSG-RA mice. Furthermore, RNA-seq and pathway analysis of the NSG-RA genome identified the IL-17 pathway as the most enriched pathway. Along with activated T-cells, B-cells also play a key role in RA as a source of rheumatoid factors and anti-citrullinated protein antibodies, and, indeed, increased frequencies of memory B-cells, plasma cells and autoantibodies were detectable in NSG-RA mice (Vinay and Kwon, 2012).

To examine to what extent NSG-RA mice reflect the human disease, histological sections of whole hind paws were examined. Histological examination focused on the synovium, a membrane-like structure consisting of a lining and a sublining layer with a variety of resident cells, of which SFs are among the most important in the progression of RA to severe disease (Wu et al., 2021). SFs contribute to the transformation of the synovial lining from a delicate structure into an invasive hyperplastic pannus, and the destruction of the joint is specifically attributed to vimentin^+^ SFs (Nygaard and Firestein, 2020). Collagen bundles, a major product of fibroblasts, can be visualized under polarized light using Sirius Red staining (Segnani et al., 2015). In NSG-RA mice, birefringent collagen bundles appear at sites of pannus formation, indicating hyperproliferative fibroblast activity. The histological analysis of NSG-RA mice, independent of additional challenge, revealed synovitis, pannus formation, cartilage erosion and focal joint destruction, indicating a nice fit of the histopathological phenotype to the disease-specific alterations described in the literature (Hayer et al., 2021). That this occurs independent of challenge suggests that RA PBMCs alone are sufficient to drive robust synovial inflammation, potentially representing a maximal inflammatory response under these conditions. The observed loss of cartilage proteoglycans, as evidenced by reduced TB staining, is consistent with hallmark features of RA pathology, whereby early depletion of cartilage matrix components contributes to progressive joint degeneration (Wakitani et al., 2002)*.* IFS confirmed the histopathological manifestations through the detection of elevated numbers of human leucocytes, including CD4^+^ T-cells, cytotoxic CD8^+^ T-cells, CD14^+^ monocytes and CD19^+^ B-cells. Additionally, the expression of mouse vimentin^+^ SFs at sites of pannus formation and CD90 (Thy1) in the hyperplastic sublining increased both *in vivo* and *ex vivo*. This suggests potential cross-talk between human leucocytes and resident mouse cells in the synovium. CD90^+^ fibroblasts proliferate within the sublining of the RA synovium and contribute to tissue destruction through multiple mechanisms (such as osteoclast differentiation/activation, IL-6 expression and fibroblast activation). This observation indicates that the NSG-RA model effectively recapitulates the cellular changes that underlie joint destruction in RA (Wiles et al., 2023).

To further validate the model, NSG-RA mice were treated either with prednisolone or infliximab, both of which are standard treatments of care in RA. Treatment with either therapeutic was sufficient to reduce the clinical frequency of paw swelling and histopathological manifestations of RA; however, the mode of action for each seemed different. Infliximab treatment led to significantly decreased plasma levels of human TNFα and IL-12 and decreased expression of *TNFA* and *IFNG* in the joints. In contrast, prednisolone only significantly reduced the plasma levels of murine Cxcl9 [an IFNγ-induced chemokine contributing to morphological and clinical features of RA (Ruschpler et al., 2003)] and decreased the expression of *Cxcl13*, *Saa1* and *IFNG*, suggesting that infliximab acts both locally and systemically in NSG-RA mice, whereas prednisolone mostly has a local effect on inflammation.

### Summary and limitations of the model

In summary, the presented NSG-RA mouse model reflects the human disease in terms of histopathological manifestations and elevated expression of markers that have previously been identified as indicators of inflammation in RA. Symptoms of NSG-RA mice improved following treatment with infliximab or prednisolone. Thus, the NSG-RA model can be used to examine inflammatory processes underlying the human disease and to validate therapeutics directed against human target molecules. In addition, compared to previously published humanized RA models, the NSG-RA model provides practical advantages, as it does not rely on engraftment of synovial tissue, CD34^+^ stem cells or fractionated PBMCs.

Because our NSG mouse models of human chronic inflammatory diseases employ engraftment with human cells, they allow for a combination of patient profiling with efficacy testing of drug candidates, potentially enabling patient stratification for designs of subsequent clinical trials and thereby contributing to risk reduction. Such models, however, suffer from inherent inter-donor variability, particularly as reflected in the induction of disease, and greater donor sampling than is presented here would be required for effective stratification using the NSG-RA model. In addition to inter-donor variability, we observed intra-donor heterogeneity among recipient mice reconstituted with PBMCs from the same RA patient. This likely reflects biological differences in reconstitution efficiency and individual host responses to stress. We consider this variability biologically relevant rather than technical noise. Although the donor represents the primary biological unit, we believe that recipient-level analyses capture relevant phenotypic differences that go beyond technical replication. Notably, an analysis using the donor as the unit of replication (Fig. S6) revealed trends consistent with those observed in the recipient-level analysis. Future studies with larger donor and recipient numbers could enable statistically robust correlation analyses between clinical disease activity and mouse phenotypes, further validating the model. We note, nevertheless, that we previously demonstrated for our NSG-UC and NSG-CD models that subgroups of mice clustered according to patient immunological profiles responded differentially to different drug treatments (Jodeleit et al., 2020; Unterweger et al., 2021a,b).

The development of inflammation in NSG-RA mice relies on the communication of resident murine fibroblasts with invading human leucocytes, and thus requires compatibility of receptors and ligands. Thus, the observed inflammation may not cover the entire inflammatory spectrum of the human disease. In future studies, alternative challenges may be considered that better reflect the chronic low-grade inflammation observed in human RA. However, such approaches would likely require prolonged study durations. Furthermore, tartrate-resistant acid phosphatase (TRAP) staining would have to be performed in future experiments to support the identification of osteoclast-driven bone erosion. For the NSG-nonRA control group, blood from a single healthy donor was used, which may limit the generalizability of the study. However, this approach follows the methodology in our established models (Palamides et al., 2016; Unterweger et al., 2021a,b; Schindler et al., 2024), where the donor's immunological background contributes to symptom development, as seen consistently across our NSG-CD, NSG-UC and NSG models for psoriasis and atopic dermatitis. Furthermore, the NSG-RA model is an acute-phase model and does not reflect the chronic human disease. Nevertheless, we believe that the NSG-RA model is likely to provide important insight into the development of the human disease and may reduce the risk of failure of novel therapeutics.

## MATERIALS AND METHODS

### Isolation of PBMCs and engraftment

60 ml of peripheral blood was collected from the arm vein of five patients with RA and one unaffected non-RA donor using trisodium citrate solution (S-Monovette, Sarstedt, Nürnberg, Germany). Patient characteristics can be found in Table S1. The isolation of PBMCs and the engraftment of mice followed a standard protocol, as previously described (Palamides et al., 2016).

### Study protocol

NSG mice were obtained from Charles River Laboratories (Sulzfeld, Germany) and kept under specific pathogen-free conditions in individually ventilated cages in a facility controlled according to the Federation of Laboratory Animal Science Association (FELASA) guidelines. Following engraftment on day 1, mice were divided into different groups. Unchallenged mice remained untreated. The other groups were challenged by intraperitoneal application of 150 µl mouse anti-type II collagen 5-clone monoclonal antibody cocktail (Arthrogen-CIA^®^, AMS Biotechnology, Abingdon, UK) on days 10 and 17 using U-100 single-use insulin syringes (Covetrus, Portland, ME, USA), followed by intraperitoneal application of 100 µl 0.05 mg/ml LPS solution (Arthrogen-CIA^®^, AMS Biotechnology, Abingdon, UK) on days 13 and 20. Mice medicated with infliximab (6 mg/kg) were injected intraperitoneally on days 12 and 19. Prednisolone was administered orally on days 12, 13, 14, 19, 20 and 21. Mice were sacrificed on day 22.

### *In vivo* hind paw swelling and weight loss

The assessment of arthritic severity during the experiment was performed according to the scoring system presented in Table S2 (Seeuws et al., 2010). The scores were summed to provide a daily total score with a maximum of 16. Animals with a daily score of >4 and/or summarized score of >7 were euthanized immediately and not taken into account. To identify differences between the treatment groups, daily scores for hind paw swelling and body weight loss were combined for each experimental group and plotted as mean±s.d. over the course of the experiment. (Minimal swelling was observed in front paws, so only hind paws were assessed for swelling.) The mice were sacrificed after 22 days, and the hind paws were dissected and photographed.

### Histopathological analysis

At necropsy, one whole hind paw of each mouse was fixed in 4% neutral buffered formaldehyde (Carl Roth, Karlsruhe, Germany) for 48 h, followed by decalcification in an acid-based standard decalcifier (Carl Roth) at 37°C for 10 days. Samples were stored in 70% ethanol and routinely embedded in paraffin, ensuring that all toes were aligned side by side in the resulting cutting planes for accurate histological analysis. Paws were cut into 4 µm transverse sections and stained with H&E (Carl Roth) or 0.05% TB (Morphisto, Offenbach am Main, Germany). Assessment of synovitis was performed according to a standardized scoring system described in Table S2, evaluating two or more interphalangeal joints per mouse (Hayer et al., 2021). Histological scores for synovitis, cartilage erosion and bone erosion were assessed per mouse, summed accordingly, and presented in separate graphs for each parameter as well as for the cumulative histological score. To ensure that the scoring reflected the most relevant pathological changes, it was based on the most severely affected joint section. Images were taken with an AXIO Observer microscope (Zeiss, Oberkochen, Germany) under 10-fold and 20-fold magnification using the software ZEN2 lite (Zeiss). In Adobe Photoshop CC, a tonal correction was used to enhance contrast within the pictures.

### Sirius Red staining

Paws were prepared as described for histopathological analysis and cut into 4 µm sections. Sirius Red staining was performed using a Picro-Sirius Red Stain Kit (ScyTek Laboratories, West Logan, UT, USA). Synovia were analysed under polarized light using a Zeiss AXIO Observer microscope and the software ZEN2 lite.

### IFS and ICC

For IFS, 4 µm hind paw sections were stained according to the protocol in Table S3 (for antibodies, see Table S4). For ICC of murine knee SFs, the medium was removed, and cells were washed twice with PBS. After fixation with 2 ml of 4% neutral buffered formaldehyde for 15 min, cells were washed with PBS, blocked 30 min in 1 ml blocking buffer (5% fetal bovine serum in PBS) and incubated overnight with the primary antibody diluted in blocking buffer at 4°C (for antibodies used, see Table S4). After two washing steps with PBS, the appropriate secondary antibody was added and incubated for 1 h at room temperature. Slides were washed three times with PBS and sealed with cover slides with mounting medium (ProLong Gold Antifade Mountant, Thermo Fisher Scientific, Waltham, MA, USA). Sections were documented under a Zeiss AXIO Observer microscope using the software ZEN2 lite.

### Isolation of splenic leucocytes

To isolate human leucocytes, at necropsy, spleens were minced, and the cells were filtered through a 70-µm cell strainer followed by centrifugation at 1400 ***g*** for 5 min and resuspension in FACS buffer (1× PBS, 2 mM EDTA, 2% fetal calf serum).

### Flow cytometry analysis

The antibodies used for labelling of human leucocytes are listed in Table S4, and the leucocytes were classified according to the markers specified in Fig. S5. All antibodies were used according to the manufacturer's instructions. Flow cytometry was performed using an Attune NxT Flow Cytometer (Thermo Fisher Scientific) and analysed with FlowJo 10.6.1-Software (FlowJo LLC, Ashland, OR, USA). Gating of cell types was performed according to Fig. S1.

### Detection of cytokines in plasma

Whole blood was collected, mixed with heparin and centrifuged at 2000 ***g*** for 10 min at 4°C. The supernatant was transferred to a fresh polypropylene tube and used immediately or stored at −80°C. Plasma was analysed using the magnet-based multiplex platform Luminex (Thermo Fisher Scientific). The Luminex assay was performed according to the manufacturer's instructions using 50 µl plasma.

### *Ex vivo* fibroblast cultivation

At necropsy, mouse knee synovium was removed and placed in an Eppendorf tube containing ice-cold PBS. Fibroblasts were isolated from NSG synovium adapting the protocol of Khalil et al. (2013) (for detailed protocol, see Table S6). After isolation, the cells were cultivated for 5 days. For H&E staining, the cells were permeabilized with 2 ml of 0.5% Tween-20 in PBS for 20 min.

### RNA extraction and cDNA synthesis

Mouse whole hind paws (skinned) were dissected and immediately submerged in RNAprotect^®^ Tissue Reagent (Qiagen, Hilden, Germany) and stored at −20°C. Paws were homogenized in QIAzol^®^ Lysis Reagent (Qiagen) with TissueLyser LT (Qiagen) followed by total RNA extraction according to the manufacturer's instructions using an RNeasy^®^ Plus Universal Mini Kit (Qiagen). For cDNA synthesis, 5 µg total RNA was used. Reverse transcription was performed in a Mastercycler gradient (Eppendorf, Hamburg, Germany) using SuperScript^®^ VILOTM Mastermix (Thermo Fisher Scientific). Samples were diluted with RNase-free water to obtain a cDNA concentration of 100 ng/20 µl reaction mix, as per the TaqMan Fast Advanced Master Mix protocol (Thermo Fisher Scientific). RNA and cDNA purity was controlled using a Nanodrop 2000 spectrophotometer (Thermo Fisher Scientific).

### Quantitative RT-PCR

According to the TaqMan Fast Advanced Master Mix protocol (Thermo Fisher Scientific) quantitative RT-PCR was performed using cDNA isolated from joint lysates using the Applied Biosystems StepOnePlus RT-PCR system (Thermo Fisher Scientific). Single-tube TaqMan gene expression assays (Thermo Fisher Scientific) included the housekeeping gene *Gapdh* (Mm99999915_g1) and the markers *TNFA* (Hs01113624_g1), *IFNG* (Hs00989291_m1), *Cxcl13* (Mm04214185_s1) and *Saa1* (Mm00656927_g1). IDs are shown in brackets. Analysis was performed using StepOnePlus™ Software v2.3 (Thermo Fisher Scientific). Relative expression values for the analysed genes were calculated as the difference between the mean cycle threshold (CT) value of the housekeeping genes and the respective analysed gene (delta CT). Relative expression is depicted as the logarithmic value of the delta CT.

### RNA-seq analysis

RNA-seq and bioinformatic analyses were performed by Novogene (Cambridge, UK). In the RNA-seq technique, single-stranded mRNAs were selectively captured and converted to cDNAs for library preparation*.* The cDNA libraries were sequenced using the state-of-the-art Illumina NovaSeq platform. *Mus musculus* (GRCm38.p3/mm10, source NCBI) and *Homo sapiens* (GRCh 38/hg38, source NCBI) were used as reference sequences for mapping. Expression values were processed to fragments per kilobase of exon per million mapped fragments (FPKM). The comparison of the unchallenged (donors, *N*=2; samples, *n*=6 in total) versus challenged (donors, *N*=3; samples, *n*=6 in total) groups was based on FPKM expression values. The ggplot2 and ggrepel R packages were used for differential expression analysis of unchallenged and challenged NSG-RA mice, with the thresholds for fold change and level of significance set at |log2 (FoldChange)|≥1 and *P*<0.05, respectively (Varet et al., 2016). KEGG enriched pathway analysis was conducted with the pathfinder package (Ulgen et al., 2019). Visualization of enriched pathways was performed with the Pathview package (Luo and Brouwer, 2013). Disease ontology analysis was performed by Novogene.

### Detection of autoantibodies

Protein microarray analysis was conducted using Austrian Institute of Technology 16k protein microarray (Luna-Coronell et al., 2016), presenting proteins recombinantly expressed in *Escherichia coli* from 15,284 human cDNA expression clones. Antibody profiling using purified IgG from plasma was conducted as described previously (Brezina et al., 2015; Rosskopf et al., 2015; Luna-Coronell et al., 2016).

In short, IgG was purified from plasma (NSG-RA, *N*=2, *n*=12 in total; NSG-nonRA, *N*=1, *n*=6 in total) using MelonGel and standardized concentrations of 0.2 mg IgG/ml, applying 450 µl per microarray for antibody profiling. Patient IgG bound onto antigens presented on microarrays was detected by a fluorescently labelled anti-human detection antibody. Background-corrected median relative fluorescent intensities (RFI) from microarray images were log2 transformed. The median normalized Log2-RFI values were then bio-statistically evaluated with BRB Array Tools Version 4.5.0 (Simon and Peng, 2003).

Mouse antibody experiments were performed with a Luminex protein bead array representing 192 recombinantly expressed proteins produced in *E. coli* cDNA clones, based on the results of the 16k protein array experiments. IgG was purified from mouse plasma with the MelonGel Spin Plate Kit according to the manufacturer's instructions. 50 µl concentration-adjusted IgG (0.05 mg/ml in PBS with 1% BSA) was used for each well and processed as previously described (Leidinger et al., 2015). Blank-corrected median fluorescence intensities were log2 transformed and subjected to bioinformatic analysis.

### Statistical analysis

Statistical analysis was performed with R (R ×64 3.6.2.ink; R Foundation, Vienna, Austria). Data presented as Cumming plots were generated using the dabestr package. Cumming plots are a new generation of data analysis with bootstrap-coupled estimation (DABEST) plots that move beyond *P*-values. These plots can be used to visualize large samples and multiple groups easily. Values were analysed for normal distribution using the Shapiro–Wilk test. Groups with the same variances were compared using a two-sided Student's *t*-test and a confidence level of 95%. If the variances were unequal, a Welch's *t*-test was performed. Groups with non-normal distribution were compared using Mann–Whitney *U*-test and a confidence level of 95%. For experiments with more than two groups, two-way ANOVA followed by Tukey's multiple comparisons test was performed for normally distributed data, whereas Kruskal–Wallis test followed by Dunn's multiple comparisons test was used for non-normally distributed data. PCA was performed using the plyr, ChemometricsWithR (Wehrens, 2011), maptools, car and rgeos packages.

### Ethical approval

Written, informed consent was given by all donors. All clinical investigations were conducted in accordance with the principles expressed in the Declaration of Helsinki. The study was approved by the Institutional Review Board of the Medical Faculty at the University of Munich (2015-22). Animal studies were approved by the animal welfare committees of the government of Upper Bavaria, Germany (ROB-55.2-2532-Vet_022088) and performed in compliance with German Animal Welfare Laws.

## Supplementary Material

10.1242/dmm.052294_sup1 Supplementary information

Table S8. All data human genome RNAseq

Table S9. All data mouse genome RNAseq

Table S10. HC-RAC Autoantibodies
