# Supplementary material for: Validation of a model of rheumatoid arthritis using mice reconstituted with patient peripheral blood mononuclear cells
Source: Dis Model Mech. 2025 Dec 29;18(12):dmm052294. doi: 10.1242/dmm.052294 (PMC12817335; doi:10.1242/dmm.052294)
Supplement: Supplementary information [file dmm-18-052294-s1.pdf]

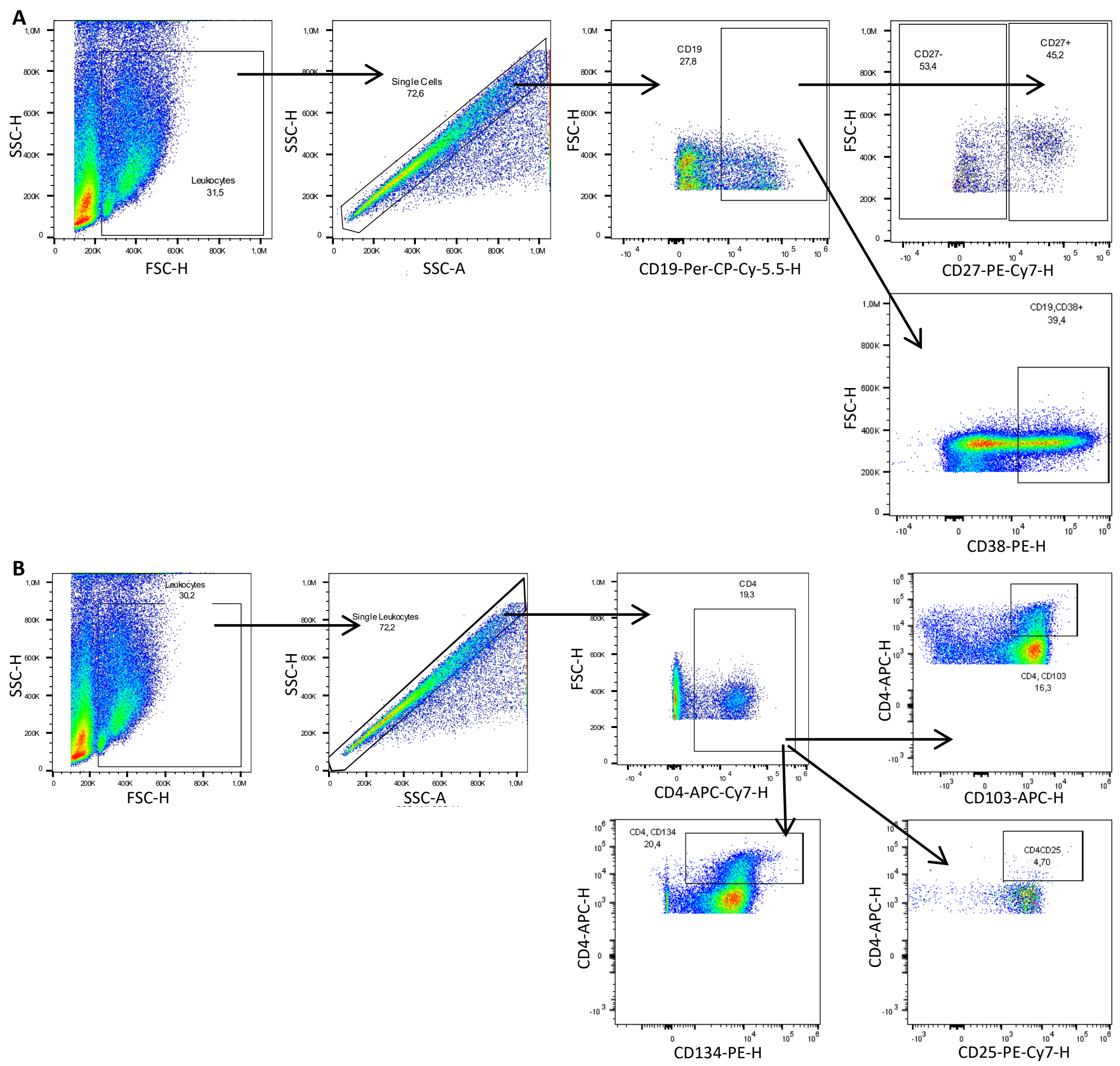

Fig. S1. Strategy of gating human leucocytes isolated from murine spleens. All cells were gated in a side scatter (SSC) and forward scatter (FSC). Single cells were isolated by plotting SSC-area vs. SSC-height. (A) Gating strategy of CD19+CD38+ plasma B cells and experienced CD19+CD27+ B cells. (B) Gating strategy of CD4+CD134+, CD4+CD103+ and CD4+CD25+ T cells.

Disease Models & Mechanisms • Supplementary information

Human Genome

NSG-RA Challenged versus NSG-RA Unchallenged

A Differentially Expressed Genes

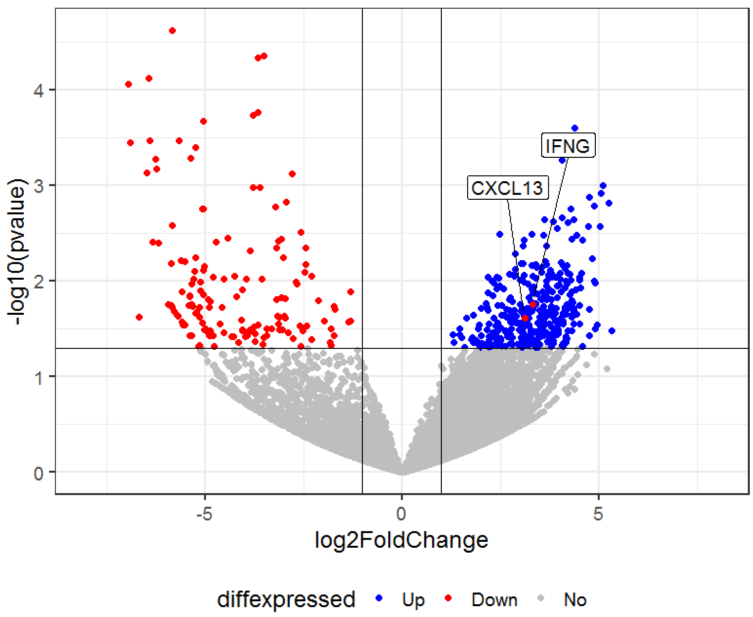

B Disease Ontology Analysis Human Genome

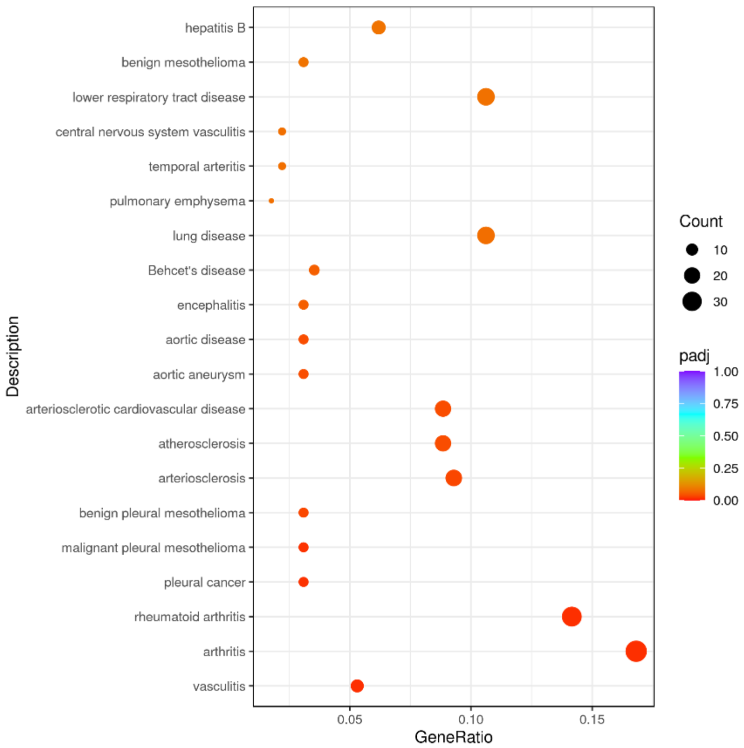

Fig. S2. RNAseq analysis of human genes. Mice were treated as described in Figure 1. RA Challenged mice (N=3, n=6 in total) and RA Unchallenged mice (N=2, n=6 in total) were compared. (A) Volcano plot with p-value<0.05 and |log2FoldChange|>1. The genes *IFNG* and *CXCL13* are highlighted in red. (B) Disease Ontology (DO) enrichment analysis.

Disease Models & Mechanisms • Supplementary information

Murine Genome

NSG-RA Challenged versus NSG-RA Unchallenged

A Differentially Expressed Genes

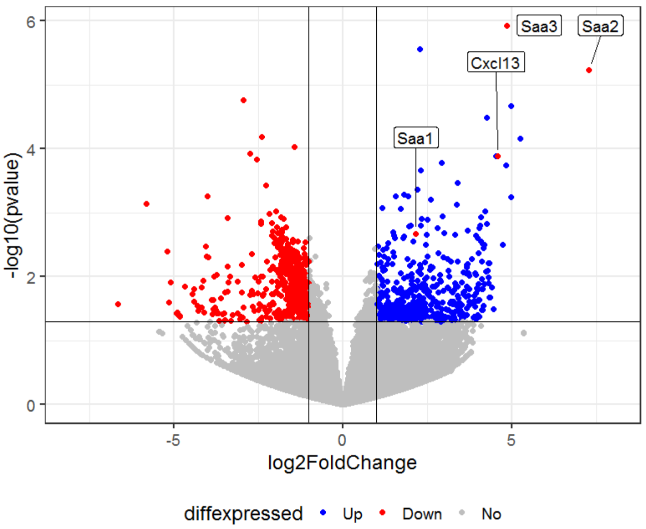

B KEGG Enriched Pathways

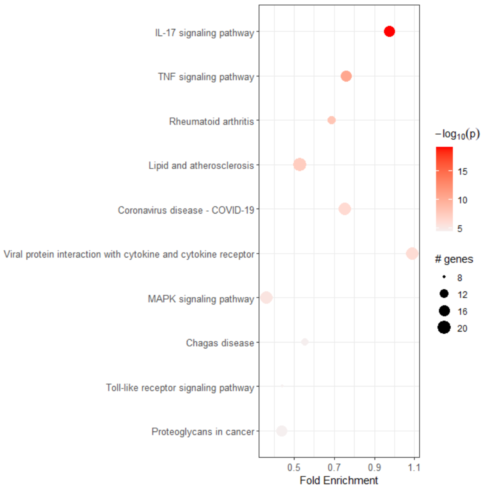

C KEGG Pathwayview

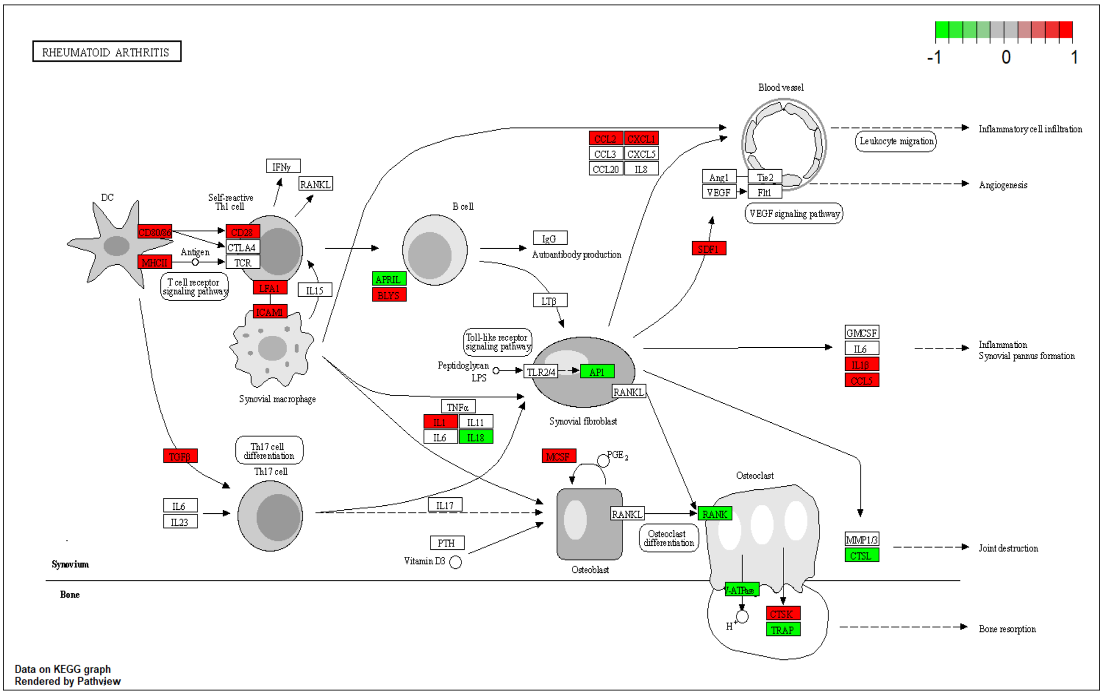

Fig. S3. RNAseq analysis of mouse genes. Mice were treated as described in Figure 1. NSG-RA Challenged mice (N=3, n=6 in total) and NSG-RA Unchallenged mice (N=2, n=6 in total) were compared. (A) Volcano plot with p-value<0.05 and |log2FoldChange|>1, The genes Saa1, Saa2, Saa3 and Cxcl13 are highlighted in red. (B) Enriched and (C) KEGG Pathwayview.

Disease Models & Mechanisms • Supplementary information

A

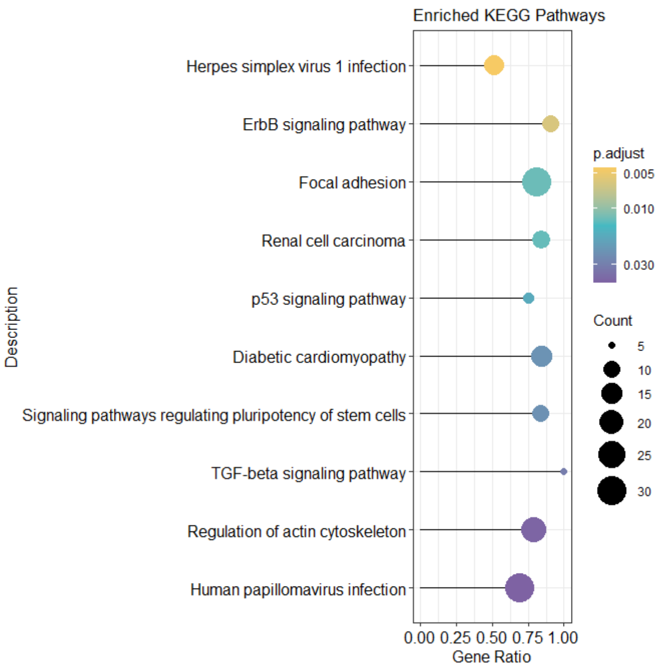

B

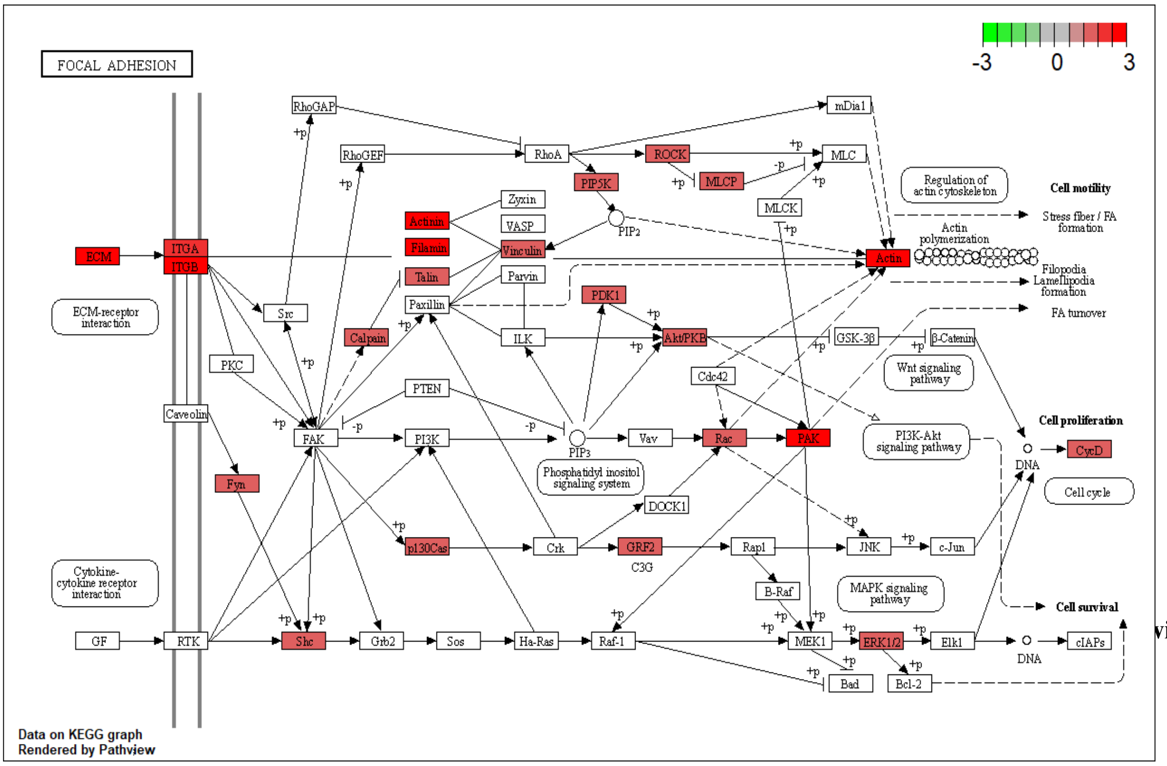

Fig. S4. Pathway analysis of autoantibodies expressed in NSG-RA mice challenged with mouse anti-type II col antibody + LPS (0,05mg/mL). (A) Enriched Kyoto Encyclopedia of Genes and Genomes (KEGG) analysis. (B) View of the enriched focal adhesion pathway. Genes encoding the proteins identified by autoantibodies are marked in red.

Disease Models & Mechanisms • Supplementary information

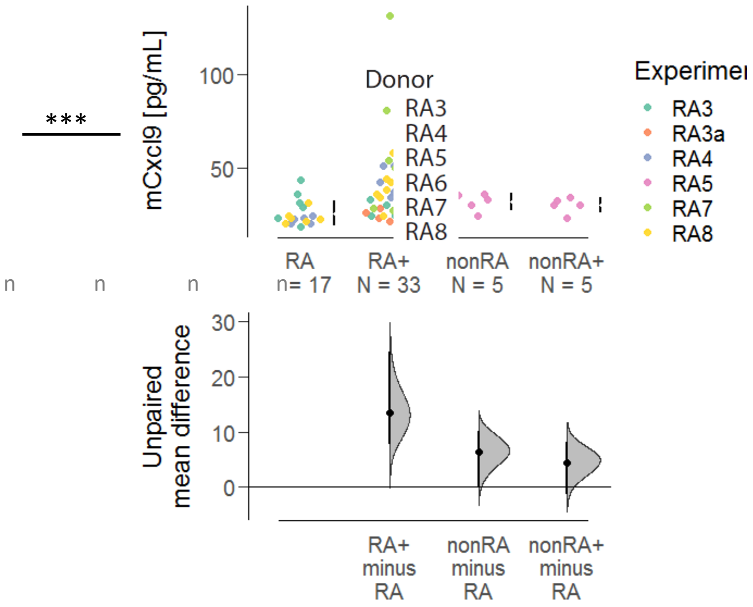

Fig. S5. mCxcl9 secretion is increased in challenged NSG-RA mice. Upon challenge, NSG-RA mice (RA+, N=5, n=33) revealed increased plasma levels of mCxcl9 in contrast to unchallenged mice (RA, N=3, n=17), or NSG-nonRA unchallenged (nonRA, N=1, n=5) or challenged (nonRA+, N=1, n=5) mice.

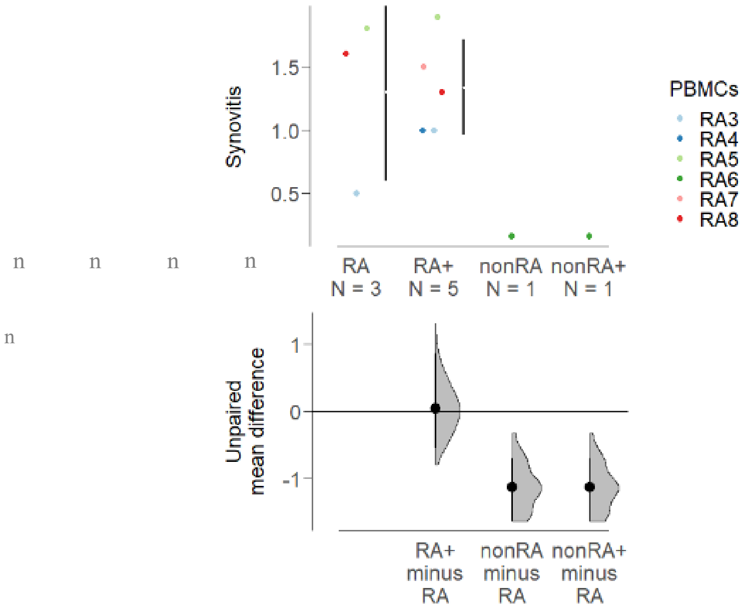

Fig. S6. Murine synovitis scores are reduced when NSG mice are engrafted with PBMCs from a nonRA donor. Engraftment with PBMCs from an RA patient (n=3 for unchallenged mice, n=5 for challenged mice) results in increased synovitis scores. In contrast, engraftment with PBMCs from a healthy donor (n=1) does no lead to such an increase in synovitis scores.

Table S1. Patient characteristics and groups defined in the animal study

Donor	Sex	Diagnosis	Medication	Fulfilled the criteria ACR/EULAR (Score)	Groups in the NSG-RA model			
					Unchallenged (n)	Challenged (n)	Prednisolone (n)	Infliximab (n)
RA3	f	RA	Etoricoxib, Hydroxychloroquin, Omeprazol	Yes (6)	6	5		
RA4	f	RA	Etanercept 50mg, Etoricoxib	Yes (6)		6		
RA5	f	RA	Arcoxia, Omeprazol	Yes (6)	5	6	6	
RA6	f	nonRA		No (0)	6	6		
RA7	m	RA	No medication	Yes (10)		6	6	6
RA8	f	RA	Arcoxia	Yes (6)	6	9		9

Table S2. Clinical activity- and histological scores for the *in vivo* experiments

**Clinical activity score**			
Swelling of the hind paw		0	no swelling
		1	minor, limited to interphalangeal joints
		2	mediocre, expansion to metatarsophalangeal joint
		3	highly, expansion to whole metatarsus
		4	serious, swelling of the whole paw including tarsotibial joint
Redness of the hind paw		0	no redness
		1	minor
		2	moderate
		3	highly
Behaviour:		0	normal
		1	reduced activity
		2	apathy
		3	ruffled fur
Body posture		0	normal
		1	intermediately hunched posture
		2	permanently hunched posture
Loss of body weight		0	0%
		1	0-5 %
		2	5-10 %
		3	10-15 %
		4	15-20 %
**Histological score**			
Synovitis	Thickening of intima	0	healthy, 1-2 cell layers
		1	2-3 cell layers
		2	multi-layered
		3	hyperplastic synovial membrane
	Synovial inflammation	0	no cellular infiltrate
		1	mild cellular infiltrate with low cell density
		2	enhanced cellular infiltrate with increased cell density

Disease Models & Mechanisms • Supplementary information

	3	maximal expanded inflammation filling all joint cavities
Bone erosion	0	healthy, intact bone surface
	1	small, superficial bone erosion at the outer surface of the cortical bone
	2	enhanced focal bone erosion
	3	massive subchondral bone erosion
Cartilage erosion	0	healthy, smooth surface of the articular cartilage
	1	minor roughening affecting 1/3 of the area
	2	moderate loss of cartilage layer affecting up to 2/3 of this area
	3	complete loss of cartilage

Table S3. Media and steps for IHC

**Media**	**Components**
Antigen retrieval buffer	10 mM trisodium citrate buffer + 0,05 % Tween, pH 6.0
Blocking buffer	1% BSA in TBS

**IHC**	
Xylol	10 min
abs. EtOH	5 min
96% EtOH	5 min
70% EtOH	5 min
aq. dest	5 min
antigen retrieval	58°C, over night
1x TBS wash	3 x 5 min
blocking	60 min, RT
1st antibody incubation	4°C, over night
1 x TBS wash	2 x 5 min
2nd antibody incubation	60 min, light protected, RT
1 x TBS wash	3 x 5 min

Table S4. Antibodies used for labelling human leucocytes (flow cytometry; FC), surface markers for immunofluorescent staining and immunocytochemistry (IFS/ICC) and Luminex ProcartaPlex™ beads. All FC antibodies were purchased from Biolegend. Antibodies for IFS/ICC were purchased from Invitrogen and Alomone. ProcartaPlex™ beads were purchased from Invitrogen.

**Surface marker**	**Color**	**Cat # RRID #**	**Clone**	**Application**	**Company**
anti-human CD4	APC/Cy7	Cat# 317417<br>RRID: AB_571946	OKT4	FC	Biolegend
anti-human CD103 (Integrin αE)	APC	Cat# 350215<br>RRID: AB_2563906	Ber-ACT8	FC	Biolegend
anti-human CD134 (OX40)	PE	Cat# 350003,<br>RRID: AB_10641708	Ber-ACT35(ACT35)	FC	Biolegend
anti-human CD25	PE/Cy7	Cat# 302611,<br>RRID: AB_314281	BC96	FC	Biolegend
anti-human CD19	PerCP/Cy5.5	Cat# 302230<br>RRID: AB_2275547	HIB19	FC	Biolegend
anti-human CD38	PE	Cat# 356603<br>RRID: AB_2561899	HB-7	FC	Biolegend
anti-human CD27	PE/Cy7	Cat# 356411<br>RRID: AB_2562257	M-T271	FC	Biolegend
Alexa Fluor™ 647 goat anti-rabbit	-	Cat# A-21244<br>RRID: AB_2535812	-	IFS/ICC 2nd AB	Invitrogen
Alexa Fluor™ 488 rabbit anti-mouse	-	Cat# A-11059<br>RRID: AB_2534106	-	IFS/ICC 2nd AB	Invitrogen
anti-mouse Vimentin	-	Cat# MA5-11883<br>RRID: AB_10985392	V9	IFS/ICC 1st AB	Invitrogen
anti-mouse/human CD90 (Thy1)	-	Cat# PA580127<br>RRID: AB_ 2747241	-	IFS/ICC 1st AB	Invitrogen
Anti-human CD4	-	Cat# 14-0049-82<br>RRID: AB_467077	-	IFS 1st AB	Invitrogen
Anti-human CD8	-	Cat# 14-0008-82<br>RRID: AB_2572848	-	IFS 1st AB	Invitrogen
Anti-human CD14	-	Cat# 14-0149-82<br>RRID: AB_467129	-	IFS 1st AB	Invitrogen
Anti-human CD19	-	Cat# 14-0190-82<br>RRID: AB_11219274	-	IFS 1st AB	Invitrogen
hIFNg ProcartaPlex™ Simplex Kit, High Sensitivity	-	EPXS010-10228-901	-	Luminex Assay	Invitrogen
hTNFa ProcartaPlex™ Simplex Kit, High Sensitivity	-	EPXS010-10223-901	-	Luminex Assay	Invitrogen
hIL17-A ProcartaPlex™ Simplex Kit, High Sensitivity	-	EPX010-12017-901	-	Luminex Assay	Invitrogen
hIL12p70 ProcartaPlex™ Simplex Kit, High Sensitivity	-	EPXS010-10238-901	-	Luminex Assay	Invitrogen

Table S5. Markers used to define human immune cell subsets from PBMCs isolated from murine spleens

Markers	Definition
CD4+CD134+	activated T-cells
CD4+CD103+	activated T-cells
CD4+CD25+	activated T-cells
CD14+CD64+	M1 monocytes
CD19+CD39+	plasma B-cells
CD19+CD27+	experienced B-cells

Table S6. Steps and media for *ex-vivo* fibroblast isolation

Media for fibroblast cultivation	
**Complete medium**	1x RPMI-1640
	10 % FBS
	1 % Penicilin-Streptomycin
**Digestion medium**	20 mL complete medium
	20 mg dispase II (Sigma Aldrich, Deisenhofen, Germany)
	10mg/mg collagenase IV (Sigma Aldrich, Deisenhofen, Germany)
	0,2 mg/ml DNase I (Sigma Aldrich, Deisenhofen, Germany)

**Step**	
**1**	replace PBS until medium becomes clear
**2**	transfer into 50mL falcon containing 10mL ice-cold 1x HBSS
**3**	37°C, 15min
**4**	wash with 10mL 1x HBSS
**5**	transfer into 20mL digestion medium
**6**	37°C, 1h15min, vortex every 15min
**7**	centrifuge at 4°C, 200 rcf, 5min
**8**	discard remaining solid tissue
**9**	centrifuge at 4°C, 200 rcf, 5min
**10**	discard supernatant
**11**	dissolve pellet in complete medium
**12**	pass cell suspension through 70 µm cell strainer
**13**	seed on coverslips (24x24mm) in 6-well-plate
**14**	add 350µl fresh complete medium to the cells
**15**	37°C, 5 % CO2, 3h
**16**	wash cells twice with 450µl HBSS
**17**	add 1200µl complete medium
**18**	37°C, 5 % CO2, 24h
**19**	replace medium after 24h

Table S7. Raw dataset table

Donor	Patient	mice gender	mice age at experiment start	Treatment	Macroscopical Score (Hind Paw Swelling)	Clinical Cummulative Score
RA3	RA	f	1m11d	Unchallenged	0	0
RA3	RA	f	1m11d	Unchallenged	0	0
RA3	RA	f	1m11d	Unchallenged	0	2
RA3	RA	f	1m11d	Unchallenged	0	0
RA3	RA	f	1m11d	Unchallenged	0	0
RA3	RA	f	1m11d	Unchallenged	0	1
RA3	RA+	f	1m11d	Challenged	0	2
RA3	RA+	f	1m11d	Challenged	0	1
RA3	RA+	f	1m11d	Challenged	0	3
RA3	RA+	f	1m11d	Challenged	3	6
RA3	RA+	f	1m11d	Challenged	0	2
RA4	RA+	f	2m23d	Challenged	0	3
RA4	RA+	f	2m23d	Challenged	1	6
RA4	RA+	f	2m23d	Challenged	2	5
RA4	RA+	f	2m23d	Challenged	1	0
RA4	RA+	f	2m23d	Challenged	1	3
RA4	RA+	f	2m23d	Challenged	2	4
RA5	RA	m	2m11d	Unchallenged	1	0
RA5	RA	m	2m11d	Unchallenged	0	2
RA5	RA	m	2m11d	Unchallenged	1	0
RA5	RA	m	2m11d	Unchallenged	1	1
RA5	RA	f	2m11d	Unchallenged	1	0
RA5	RA+	m	2m11d	Challenged	2	2
RA5	RA+	m	2m11d	Challenged	0	2
RA5	RA+	m	2m11d	Challenged	3	9
RA5	RA+	m	2m11d	Challenged	3	7
RA5	RA+	f	2m11d	Challenged	2	4
RA5	RA+	f	2m11d	Challenged	1	7
RA5		m	2m11d	Prednisolone	0	3
RA5		m	2m11d	Prednisolone	0	4

Disease Models & Mechanisms • Supplementary information

RA5		m	2m11d	Prednisolone	0	1
RA5		m	2m11d	Prednisolone	1	3
RA5		f	2m11d	Prednisolone	2	2
RA5		f	2m11d	Prednisolone	1	3
RA6	nonRA	f	3m8d	Unchallenged	0	1
RA6	nonRA	f	3m8d	Unchallenged	0	1
RA6	nonRA	f	3m8d	Unchallenged	0	0
RA6	nonRA	f	3m8d	Unchallenged	0	0
RA6	nonRA	f	3m8d	Unchallenged	0	4
RA6	nonRA	f	3m8d	Unchallenged	0	0
RA6	nonRA+	f	2m18d	Challenged	0	3
RA6	nonRA+	f	2m18d	Challenged	1	1
RA6	nonRA+	f	2m18d	Challenged	0	0
RA6	nonRA+	m	2m18d	Challenged	0	2
RA6	nonRA+	m	2m18d	Challenged	0	3
RA6	nonRA+	m	2m18d	Challenged	0	2
RA7	RA+	m	2m17d	Challenged	2	3
RA7	RA+	m	2m17d	Challenged	1	2
RA7	RA+	m	2m17d	Challenged	1	2
RA7	RA+	f	2m17d	Challenged	2	2
RA7	RA+	f	2m17d	Challenged	2	4
RA7	RA+	f	2m17d	Challenged	2	3
RA7		m	2m17d	Prednisolone	1	4
RA7		m	2m17d	Prednisolone	0	3
RA7		m	2m17d	Prednisolone	0	3
RA7		f	2m17d	Prednisolone	1	6
RA7		f	2m17d	Prednisolone	0	3
RA7		f	2m17d	Prednisolone	1	6
RA7		m	2m17d	Infliximab	1	1
RA7		m	2m17d	Infliximab	0	2
RA7		m	2m17d	Infliximab	2	6
RA7		f	2m17d	Infliximab	1	2
RA7		f	2m17d	Infliximab	1	4

Disease Models & Mechanisms • Supplementary information

RA7		f	2m17d	Infliximab	2	7
RA8	RA	m	1m25d	Unchallenged	1	2
RA8	RA	m	1m25d	Unchallenged	1	0
RA8	RA	m	1m25d	Unchallenged	1	0
RA8	RA	f	1m25d	Unchallenged	1	0
RA8	RA	f	1m25d	Unchallenged	0	1
RA8	RA	f	1m25d	Unchallenged	0	0
RA8	RA+	m	1m25d	Challenged	1	2
RA8	RA+	m	1m25d	Challenged	1	4
RA8	RA+	m	1m25d	Challenged	1	5
RA8	RA+	f	1m25d	Challenged	1	3
RA8	RA+	f	1m25d	Challenged	4	8
RA8	RA+	f	1m25d	Challenged	4	7
RA8	RA+	m	1m25d	Challenged	4	7
RA8	RA+	m	1m25d	Challenged	2	4
RA8	RA+	m	1m25d	Challenged	2	5
RA8		m	1m25d	Infliximab	0	1
RA8		m	1m25d	Infliximab	0	3
RA8		m	1m25d	Infliximab	2	3
RA8		f	1m25d	Infliximab	0	4
RA8		f	1m25d	Infliximab	0	1
RA8		f	1m25d	Infliximab	2	8
RA8		f	1m25d	Infliximab	0	4
RA8		f	1m25d	Infliximab	0	4
RA8		f	1m25d	Infliximab	4	8

Disease Models & Mechanisms • Supplementary information

Hind Paw Swelling Clinical	Histological Score	Synovitis	Bone Erosion	Cartilage Erosion	Lining Layer Hyperplasia	CD45	CD4CD25
0	1	1	0	0	0	3,54	11,2
0	0	0	0	0	0	3,1	15,4
0	3	1	0	1	1	2,92	10,3
0	2	1	0	0	1	9,73	27,9
0	2	0	0	1	1	3	10,1
0	1	0	0	1	0	5,1	17,4
0	5	2	0	1	2	12,7	13,6
0	0	0	0	0	0	8,37	25,9
0	0	0	0	0	0	13,2	19
5	4	2	0	1	1	17,8	12,6
0	2	1	0	1	0	8,69	14,1
0	1	0	0	0	1	42	22,4
3	6	2	0	1	2	38,7	17,7
2	2	1	0	0	1	44	27,6
0	2	1	0	0	1	30,9	11,7
0	3	1	0	0	1	37	21
0	3	1	0	1	1	20	6,32
0	5	2	1	1	1	19,6	5,97
0	5	2	0	1	1	17,3	3,73
0	4	2	0	0	1	33,7	15,9
0	5	2	0	1	1	19,3	3,22
0	4	1	0	1	1	21,6	4,7
0	5	2	1	0	2	20,3	2,63
0	5	2	0	1	1	19,4	2,84
7	2	1	0	1	0	31,8	12,8
5	6	3	0	1	2	24,7	9,24
1	2	1	0	1	0	20,9	8,37
0	5	2	1	0	2	30,8	10,4
0	2	1	0	0	1	25,7	7,27
4	2	1	0	0	1	26,8	7,32

Disease Models & Mechanisms • Supplementary information

0	3	1	1	0	1	19,5	7,49
0	3	1	0	1	1	36,5	11,9
1	6	2	1	1	2	32,4	9,71
0	3	1	0	1	1	40,6	19,1
0	2	1	0	0	1	14,6	7,65
0	1	0	0	0	1	14,5	11,8
0	0	0	0	0	0	16,1	10
0	1	0	0	0	1	12,5	8,67
3	0	0	0	0	0	15,2	5,52
0	0	0	0	0	0	14,9	5,89
1	0	0	0	0	0	18,5	6,13
0	1	0	0	0	1	16,7	8,58
0	0	0	0	0	0	15,4	8,42
0	0	0	0	0	0	14,5	6,95
0	2	1	0	0	1	17,2	3,53
0	0	0	0	0	0	14,3	5,96
0	5	3	0	0	2	12,3	12,6
0	1	1	0	0	0	13,7	4,9
0	7	2	1	2	2	13,9	7,39
0	3	1	0	1	1	12,7	3,24
0	1	1	0	0	0	19,5	11,9
1	3	1	0	0	2	17,1	10,8
3	0	0	0	0	0	13,3	13,6
0	1	0	0	0	1	11,7	6,67
0	2	1	0	0	1	11,7	8,95
4	2	1	0	0	1	14,4	6,76
2	2	1	0	0	1	20,9	12,1
5	0	0	0	0	0	17,6	7,99
0	0	0	0	0	0	14,9	9,59
0	1	0	0	0	1	18,2	9,67
4	3	2	0	0	1	18,5	7,42
0	5	2	0	1	2	13,9	6,27
0	1	0	0	0	1	13,5	11,8

5	0	0	0	0	0	21	13,2
0	3	1	0	1	1	18,9	7,11
0	4	2	0	0	2	19	5,88
0	3	1	1	0	1	28,1	9,94
0	5	2	0	1	2	28,8	11
0						17	11,8
0	5	2	0	1	2	17,7	9,85
0	4	2	0	0	2	29,3	15,9
0	4	2	0	0	2	15,6	5,78
0	5	2	0	1	2	29,4	12,8
0	1	0	0	0	1	15,7	13,4
5	4	1	1	1	1	29,8	7,59
4	2	1	0	0	1	24,5	8,81
7	6	2	1	1	2	17,4	7,42
1	3	1	0	0	2	23,1	7,67
1	2	1	0	0	1	13,1	5,04
0	2	1	0	0	1	34	15,1
0	3	1	0	1	1	12,3	4,97
0	0	0	0	0	0	11,5	3,99
0	4	2	0	0	2	20,8	10,5
0	0	0	0	0	0	12,7	4,02
3	0	0	0	0	0	17,1	8,87
0	0	0	0	0	0	12	10,2
0	1	0	0	0	1	23,5	13,3
6	6	2	1	1	2	22,6	10,5

CD4CD103	CD4CD134	CD19CD27	CD19CD38	CD14CD64	Plasma hIFNg	Plasma hTNFa	Plasma hIL12p70	Plasma IL17-A	mCXCL13 lg-dCT
12,1	20,8	70,3	15,1	17,4	374	15	6	12	0,000338853
16,4	26,2	68,4	16,4	21,1	64.5	16	5	12	0,080507808
11,3	20,8	66,9	9,34	19,4		53	6	14	0,009323177
28,4	35,8	73,9	44,4	25,9	11820	18	7	21	0,003223618
10,9	18,3	60,4	9,52	13,2	4238	32	5	20	0,034203305
18,1	26,2	70,4	35,5	20,9	2605.5	23	5	15	0,001500276
14,3	20,8	75,5	51,4	16,7					
27,5	39,4	67,3	44	25,4					
19,8	27,4	68,1	42,3	19					
13,7	20,4	55,9	13,9	6,84					
14,7	21,1	44,1	40	10,4					
23,1	27	71,8	30,5	23,7	6480.5	37	6	44712	0,346121126
18,2	20,9	67,4	29,5	19,2	8749.5	37	14	33	0,091004749
27,9	28,3	75	27,4	17,1	6015	33	6	23	0,058094929
12,2	16,5	70,3	30	14,4	1362	23	6	18	0,011687545
21,5	24,2	65,9	29,1	23,8	1417	22	6	15	1,275945152
6,65	10,1	48,8	16,4	17	190	20	5	11	384,0229377
6,57	5,31	61,6	5,43	4,7	1196		9	16	0,005500783
4,14	3,22	53,5	3,44	5,74	438		9	16	0,001074441
16,5	14,4	62,4	19,7	8,56	1622		9	20	0,018271447
3,57	2,78	59,1	4,77	4,57	57		8	17	0,003341098
5,19	4,08	59,2	6,35	3,59	126,5		8	17	0,00230623
2,98	2,23	53,5	3,35	5,09	283		9	16	0,006660314
3,18	2,4	57,8	4,05	10,4	41		8	15	0,006556263
13,5	11,1	53,1	17,7	16,2	3167		12	32	0,005820987
9,63	8,24	48,8	11	12,2	3641		11	17	0,006821993
8,82	7,68	58,8	9,51	7,34	1050,5		10	21	0,028071076
10,9	9,63	60,1	17,9	8,95	6056		11	31	0,003541831
7,69	6,53	58,9	18,3	7,37	134		9	17	0,013996144
7,93	6,34	65	23,2	12,7	604		12	37	0,012214811

Disease Models & Mechanisms • Supplementary information

8,06	6,64	55,9	14,7	17,3	34		8	18	0,005775186
12,5	10,6	54,6	14	12,2	2857		10,5	20	0,004361144
10,2	8,84	56	9,71	10	2697		9	31	0,028871424
19,9	17,3	85,2	40,2	12	5298		13	37	
6,03	2,8	27,1	4,81	12,6	13	19	6	9	0,000408849
9,37	3,85	27	4,47	21,8	36	20	5	10	0,020613354
8,05	4,38	25,6	3,68	16,5	76	19	6	11	0,017035399
7,31	3,66	28,5	5,8	14,6	110	18	6	10	0,148242282
5,1	2,01	22,5	3,28	7,98	11	17	6	10	0,077124003
4,9	2,14	26,7	4,91	10,6	57	16,5	6	11	0,048421196
4,9	2,51	21,4	2,21	10,5	11	22	6	9	0,037846564
6,85	3,91	21	1,98	13,5	11	18	6	11	0,095786796
6,56	4,34	22,3	2,54	11,6	11	15	5	9	0,058237116
5,63	3,3	17,6	1,25	14,6	13	16	6	11	0,019278503
3,13	1,31	17,3	1,07	6,16	11	15,5	6	44691	0,032944978
5,26	1,76	23	1,89	8,11	126.5	17	6	10	0,036905429
13,2	9,98	55,4	3,71	25,2	6440	61	44687	44694	0,172528967
5,29	4	46,2	1,94	15,3	16	60	7	12	0,013180902
7,86	6,09	48,7	2,94	16,6	330.5	21	7	12	0,17171454
3,58	2,53	47,6	4,74	12,2	15	34	7	15	0,005239341
12,4	9,75	44,6	1,74	12,2	2728	39	7	16	0,074884099
11,4	8,82	65,3	5,28	23,8	16	14	6	10	0,295084611
14,5	10,7	56,8	3,37	29,2	917	36	7	11	0,005290417
7,19	5,33	66,8	7,01	21,7	29	16	6	11	0,095309428
9,42	7,4	58,4	2,94	19,2	798.5	18	6	11	0,159364781
7,27	5,4	47,9	1,67	16,1	14	15	6	11	0,010063742
12,6	10	50,3	8,13	17,8	1972.5	23	6	12	0,017042923
8,42	6,6	44,1	2,52	14	1099.5	17	6	10	0,004822475
10,1	7,81	49,7	1,88	19,2	611	15	6	12	0,110939762
10,2	7,77	56,5	2,93	20,9	12	13	6	11	0,003731411
7,63	6,37	55,7	2,51	11,2	147	16	7	11	0,014502434
6,83	4,86	50,1	1,81	20,7	109	18	6	11	0,300477205
12,6	9,23	57,5	2,92	31,5	10	12	5	9	0,92421296

Disease Models & Mechanisms • Supplementary information

14,1	10,4	47,1	9,4	19,6	3818	16	6	15	0,012810939
6,15	9,15	69,2	81,3	13,4	822	17	7	11	
5,12	8,32	69,7	57,5	12,4	936	16	8	13	0,000648685
7,15	15,5	67,5	49,9	13,4	7681	31	6	17	0,000299723
9,69	15,8	66,3	49,9	16,7	338	19	7	12	0,005617319
8,83	14	75,3	82,8	20,1	54	18	6	10	0,002565177
8,12	12,8	67,9	73,2	19,8	585	20		12	0,001769399
11,7	23,8	67,1	55,7	22,1	216	22	6	13	1,392248745
4,8	8,48	73,1	81,6	12,8	14	14	7	10	0,92785275
11,6	20,1	63,9	53,6	22,3	385	20	6	12	0,116021775
12,8	22,3	71	76,6	14,6	11	16	7	11	0,005892548
6,31	8,84	66,1	53,3	17,1	303	18	7	12	0,606372589
6,68	13,9	72,5	46,5	11,7	260	19	6	12	0,166506513
7,63	11,2	69,4	73,3	12,4	109	20	7	12	0,008727489
7,38	11	62,7	45	16,3	75	13	7	13	0,188065835
5,05	6,49	70,1	79,9	12,6	12	13	6	10	0,203953232
14,3	25,3	69,8	56,4	21,4	597	21	6	12	0,241375686
4,91	7,21	72,6	80,9	11	11	13	6	11	0,10112957
3,62	5,25	70,7	81,2	10,3	9	13	6	44691	0,234220165
8,71	16,4	73,5	66,6	19	16	12	6	10	0,592065141
3,75	6,21	65,5	43,6	8,11	12	14	6	12	0,036547822
7,35	12,8	69,6	65,9	18,4	31	13	6	11	3,29441E-06
9,09	13,4	72,7	83	21,5	17	13	5	13	1,373211518
11,9	20,4	69,9	57,1	18,7	43	13,5	44686	12	0,626494871
8,79	15,4	67,1	50,7	16,9	46	40	6	12	0,018191448

hIFNg lg-dCT	mSaa1 lg-dCT	hTNFa lg-dCT
1,491E-05	0,0024182	1,491E-05
0,003343	0,0039334	0,0007596
0,000362	0,0170766	0,0001204
0,0002944	0,0312386	0,0003368
0,0002444	0,0034375	0,0009191
2,675E-05	0,0010062	2,582E-05
0,0062722	0,0219765	0,001901
0,0021325	0,0085904	0,0003752
0,0022429	0,0062692	0,0007859
6,33E-05	0,0065512	7,734E-06
0,0007418	0,0007418	0,0007418
6,881E-05	0,0007202	6,881E-05
6,343E-05	0,0057116	0,0011847
0,0002124	0,0002124	0,0002124
3,818E-05	0,0018897	3,818E-05
4,489E-05	0,0003192	0,0003684
3,366E-05	0,0106047	6,59E-06
0,0004225	0,0033305	3,366E-05
0,0001498	0,0032679	0,0003925
0,0012446	0,0069719	0,0001266
0,0030727	0,0113067	0,0012032
5,303E-05	0,001427	0,0006105
9,988E-05	0,0092362	0,0008082
0,0002958	0,0067123	0,0001608

6,538E-06	0,0047524	3,67E-05
0,0005419	0,0040013	6,538E-06
3,093E-11	0,0208359	0,0027607
0	5,267E-05	0
0	0,0001441	0
0	0,0009355	0
0	0,0025072	0
0	0,0001993	0
	0,0011729	0
0	1,472E-05	0
0	0,0058599	0
0	3,167E-05	0
0	0,0005652	0
0	0,0008695	0
0	0,0050581	0
0,0004196	0,0818368	0,0005284
3,276E-05	0,0047918	3,276E-05
0,0002722	0,0393736	7,672E-06
1,237E-05	0,0041647	1,237E-05
0,0025855	0,0469144	0,0001686
4,15E-06	0,0946412	1,434E-05
8,739E-05	0,0052325	4,475E-06
2,956E-06	0,0395296	2,956E-06
0,0002264	0,0306472	0,0001138
0,0001326	0,0010779	0,0001326
0,0001032	0,0016345	1,505E-05
0,0004371	0,0013019	0,000144
0,0003102	0,0949801	0,0001728
2,807E-06	0,0017275	2,807E-06
5,08E-05	0,0088951	3,209E-05
0,0001064	0,1571233	0,0001038
1,481E-05	0,4985683	1,481E-05

Disease Models & Mechanisms • Supplementary information

0,0004543	0,0064885	0,0001547
3,81E-05	0,001567	4,912E-05
0,0008134	0,0081912	0,000354
0,000176	0,0008521	0,0003245
5,628E-06	0,0011548	5,628E-06
6,327E-06	0,0023387	6,327E-06
0,0007791	0,2269399	0,0006586
6,956E-06	0,2064398	6,956E-06
0,0003341	0,103471	5,786E-05
1,057E-05	0,0059643	1,057E-05
0,0005594	0,2300145	0,0001653
0,000203	0,0377641	0,000191
4,283E-05	0,0025386	4,878E-06
0,0003109	0,0291093	5,935E-06
0,0010514	0,0642386	0,0001863
0,0008063	0,0583641	0,0001674
8,959E-06	0,0613452	8,959E-06
2,943E-05	0,0380219	2,943E-05
9,054E-05	0,1788872	6,237E-05
0,0001145	0,0009177	0,0001145
2,403E-05	0,0716231	3,294E-06
7,812E-06	0,0871332	7,812E-06
0,0001282	0,1703643	5,076E-06
0,0003167	0,0017439	8,116E-05

Table S8. All data human genome RNAseq

Available for download at
https://journals.biologists.com/dmm/article-lookup/doi/10.1242/dmm.052294#supplementary-data

Table S9. All data mouse genome RNAseq

Available for download at
https://journals.biologists.com/dmm/article-lookup/doi/10.1242/dmm.052294#supplementary-data

Table S10. HC-RAC Autoantibodies

Available for download at
https://journals.biologists.com/dmm/article-lookup/doi/10.1242/dmm.052294#supplementary-data
